# Phylogenetic Relationship Among Wild and Cultivated Grapevine in Sicily: A Hotspot in the Middle of the Mediterranean Basin

**DOI:** 10.3389/fpls.2019.01506

**Published:** 2019-11-26

**Authors:** Roberto De Michele, Francesca La Bella, Alessandro Silvestre Gristina, Ignazio Fontana, Davide Pacifico, Giuseppe Garfi, Antonio Motisi, Dalila Crucitti, Loredana Abbate, Francesco Carimi

**Affiliations:** Institute of Biosciences and Bioresources (IBBR), National Research Council of Italy (CNR), Palermo, Italy

**Keywords:** grapevine, *Vitis vinifera* subsp. *sativa*, *Vitis vinifera* subsp. *sylvestris*, domestication, SSR

## Abstract

Grapevine (*Vitis vinifera* ssp. *sativa*) is a perennial crop especially important for wine and fruit production. The species is highly polymorphic with thousands of different varieties selected by farmers and clonally propagated. However, it is still debated whether grapevine domestication from its wild ancestor (*V. vinifera* ssp. *sylvestris*) has been a single event or rather it occurred on multiple occasions during the diffusion of its cultivation across the Mediterranean. Located in the center of the Basin, Sicily is its largest island and has served as a hotspot for all civilizations that have crossed the Mediterranean throughout history. Hundreds of unique grapevine cultivars are still cultivated in Sicily and its surrounding minor islands, though most of them are menaced by extinction. Wild grapevine is also present with isolated populations thriving along riverbanks. With the aim to evaluate the phylogenetic relationships among Sicilian varieties, and to assess the possible contribution of indigenous wild populations to the genetic makeup of cultivated grapevine, we analyzed 170 domestic cultivars and 125 wild plants, collected from 10 different populations, with 23 SSR markers. We also compared our data with published dataset from Eurasia. Results show that Sicilian wild populations are related to the cultivated Sicilian and Italian germplasm, suggesting events of introgression and/or domestication of local varieties.

## Introduction

Grapevine (*Vitis vinifera* L.) is one of the most widespread and economically important perennial crops on the planet. It was estimated that in 2016 the world vineyard area was 7.4 million hectares, with a production of 76 million tons of fresh grapes and 269 million hectoliters (mhl) of wine (http://www.oiv.int/). The cultivation of the domesticated grape (*V. vinifera* L. subsp. *sativa* (DC.) Hegi) is believed to have started at least 7,000–8,000 years ago from its wild progenitor (*V. vinifera* L. subsp. *sylvestris* (Gmel.) Hegi (McGovern, 2003). Archaeological and historical studies evidenced that the primary center of domestication of the grapevine is located between the Near East (Zohary et al., 1996) and the Transcaucasian region (Olmo, 1976), then the grapevine spread around the Mediterranean, following the main civilizations (Carthaginians, Etruscans, Phoenicians, Greeks, and Romans) (McGovern, 2003). During its spreading across the Western Mediterranean regions, the grapevine increased its genetic variability due to the contribution of multiple genetic pools and progressive human selection (Bacilieri et al., 2013). Different studies support the presence of secondary domestication centers, where spontaneous hybridizations among cultivated forms and local wild plants, or direct selection, generated the pattern of the modern Western European cultivars (Grassi et al., 2003; Arroyo-García et al., 2006; Myles et al., 2011; De Andrés et al., 2012; Riaz et al., 2018). Nowadays, more than 6,000 cultivated varieties are recorded (Lacombe et al., 2013; in OIV, 2017). The genotypes of the cultivated vine are highly heterozygous and most of the modern cultivars are hermaphrodite, self-fertile, and easily crossed (This et al., 2006). Several authors reported a high genetic diversity within the subsp. *sativa*, although it was recently demonstrated that such variability is included within a complex network of close pedigree relationships, derived by crosses among elite cultivars (Myles et al., 2011).

The domestic and the wild vine can be distinguished by morphological differences concerning leaves, flowers, and fruits, although in most cases the distinction of wild grape is hampered by the gene flow between the two subspecies (Di Vecchi-Staraz et al., 2009). The wild grapevine is a dioecious liana that grows in northern Africa, Europe, and the Near and Middle East, in areas between 30° and 50° north latitude. In central and eastern Europe, it thrives in mixed deciduous forests in correspondence with warmer (southern exposure) and humid (valleys of the Rhine, the Loire, the Rhone, the Danube, etc.) microclimates, while in the Mediterranean region it mainly participates in the riparian woodlands (pure or mixed populations dominated by poplars, willows, elms, ash trees, alders in areas with shallow water; pioneer shrub communities with tamarisks and oleanders along the middle-terminal section of the streams; mixed stands with holm and downy oak; shrubby mantle assemblages). At present time wild grapevine has become rather rare due to several forms of human disturbance, such as habitat destruction and fragmentation, silvicultural practices, diffusion of pathogens (e.g., oïdium, phylloxera, mildew, and viruses), improper management of natural environment, and hybridization with domestic forms (Arrigo and Arnold, 2007; Zecca et al., 2010; Garfi et al., 2013; Pacifico et al., 2016; Arnold et al., 2017). Gene flow between wild and cultivated grapevines was confirmed in several countries such as Spain (Arroyo-García et al., 2006; De Andrés et al., 2012), Italy (Zecca et al., 2010) and Georgia (Ekhvaia et al., 2014). In the last years, molecular methods based on the use of microsatellite (SSR) (This et al., 2004; Grassi et al., 2008; Carimi et al., 2011; Lacombe et al., 2013; Emanuelli et al., 2013) and, more recently, on single-nucleotide polymorphism (SNP) markers (Salmaso et al., 2004; Myles et al., 2011; Emanuelli et al., 2013; Laucou et al., 2018; De Lorenzis et al., 2019), as well as on genome sequencing (Zhou et al., 2017) allowed not only to improve the discrimination between wild and cultivated populations, but also to study the relationships among different cultivated varieties and wild accessions.

In Italy, grapevine cultivation is reported since the second half of the 2nd millennium BCE, starting from the Southern regions and then moving northward in the second part of the 1st millennium (Hopf, 1991; Forni, 2012). However, the recent discovery of a large storage jar containing tartaric acid could date back to the Copper Age (early 4th–3rd millennium BCE) the origin of winemaking in Sicily (Tanasi et al., 2017). Sicily and its satellite islets host a rich vascular flora and due to its central position in the Mediterranean, the island has played and still plays a key role in connecting both plant and human populations of neighboring Mediterranean countries. Among plant species *V. vinifera* subsp. *sylvestris* is also present in the region with isolated populations mainly thriving along riverbanks (Garfì et al., 2013). Moreover, the island boasts a very ancient and rich tradition of viticulture practices and more than 70 different cultivars have been found in mainland Sicily (Carimi et al., 2010; Carimi et al., 2011). In addition, the Sicilian minor islets have recently emerged as a hotspot of genetic diversity for grapevine. Genetic analyses of this germplasm showed that at least 75 different genetic profiles are present in the Aeolian and Pelagie archipelagos, and the isles of Pantelleria and Ustica. Most of these genetic profiles (39) were not listed in national and international grapevine databases (Gristina et al., 2017). Such notable variety may have originated from domestication of wild autochthonous grapevines as well as from introduction of domesticated varieties from different regions during various historical periods.

In order to provide meaningful insights into grapevine evolution and domestication in the Mediterranean Basin, in this work, we compared the unique plant material constituted by the relict populations of Sicilian wild grapevine to the cultivated local germplasm, as well as to grapevine accessions from Western Europe and Central Asia. To evaluate the phylogenetic relationships among Sicilian varieties, and to assess the possible contribution of indigenous wild populations to the genetic makeup of cultivated grapevines, we analyzed with 23 nuclear SSR markers 170 local cultivars (*V. vinifera* spp. *sativa*), from the main Island and surrounding archipelagos, and 125 wild plants (*V. vinifera* spp. *sylvestris*) collected from 10 different Sicilian populations.

## Materials and Methods

### Plant Materials, Study Sites, and Sampling

The list of cultivated and wild accessions analyzed in this study includes all the cultivars that had already been described as traditionally cultivated in Sicily and its minor islands (Carimi et al., 2010; Carimi et al., 2011; Gristina et al., 2017) and all the natural populations identified so far (Garfì et al., 2013), plus other cultivated and wild accessions collected in subsequent surveys.

Most part of the grapevine cultivars and the wild germplasm collected in the field is conserved in the germplasm repository for perennial plants by the Institute of Biosciences and BioResources of the National Research Council of Italy (CNR-IBBR) located in Collesano district (province of Palermo), Italy (37°59'19.9"N, 13°54'55.8"E, 80 m a.s.l.).

**Figure 1:**
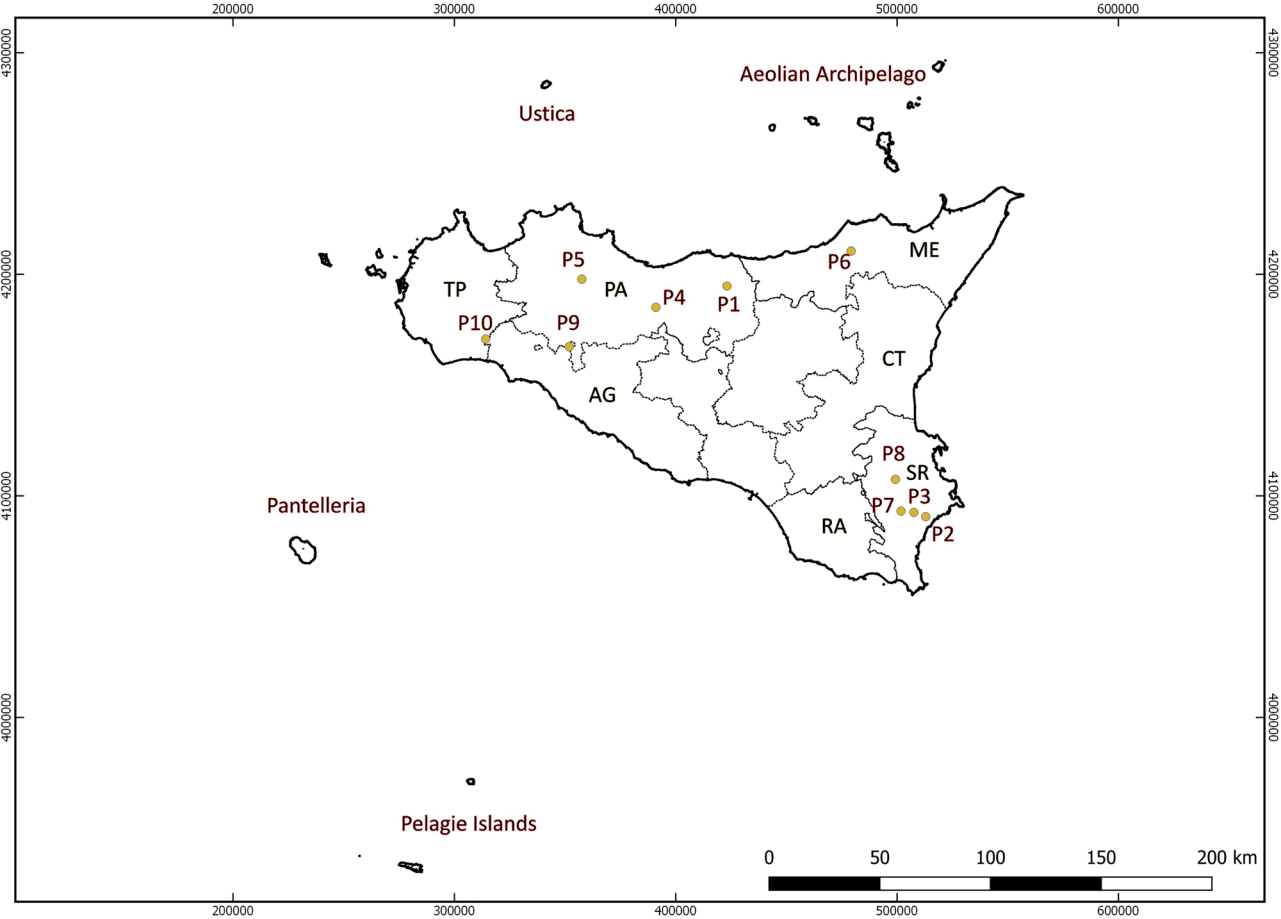
Map of Sicily indicating the collection sites of wild grapevine.

Wild *Vitis* germplasm was collected during several surveys between 2007 and 2016 in the main mountainous and protected areas of Sicily (**Figure 1**). Considering the morphologic resemblance of wild and cultivated grapevines, in order to reduce as much as possible the risk of collecting plants deriving from naturalized grapevine cultivars or rootstocks, the sampling strategy for *V. sylvestris* was based on the main differentiating reference traits used to distinguish wild grapevines from domesticated ones (Olmo, 1976; Garfì et al., 2013). Morphological data acquired *in situ* were integrated by 3–5 years of ampelographic studies carried out on grafted plants at the CNR-IBBR germplasm repository. In total, we collected 131 plants (**Supplementary Table S1**) from 10 different populations (**Supplementary Table S2**). Following molecular screening, four clones (3076 = 3074-P6, 3045 = 3058 = 3059 P4, 3139 = 3140-P10) and two feral forms (3109-P8, 3143-P10) were excluded from further analysis (final wild samples analyzed = 125).

Cultivated germplasm was collected between 2006 and 2017 directly from old vineyards. The cultivated accessions were selected following the indications of farmers and labeled for subsequent analysis and plant propagation. In total, 104 accessions were collected from Sicily and 66 from the surrounding minor islands (**Table 1**).

**Table 1:** List of cultivated and wild accessions of *Vitis vinifera* (295) grouped into groups based on their geographic origin and analyzed by 23 SSR markers. The number of samples for each group is presented in brackets.

*V. vinifera* subsp. *sativa* (170)	*V. vinifera* subsp. *sylvestris* (125)
Sicily main Island (104)	**P1** Castelbuono, Madonie Mts., Palermo (16)
Agrigento (8)	**P2** Cava Grande Cassibile, Iblei Mts. Syracuse (14)
Catania (19)	**P3** Cava Sturia, Iblei Mts. Syracuse (4)
Messina (9)	**P4** Riserva Boschi Favara and Granza, Torto Valley Palermo (16)
Palermo (22)	**P5** Bosco della Ficuzza, Sicani Mts. Palermo (9)
Ragusa (7)	**P6** Stretta di Longi, Nebrodi Mts. Messina (12)
Syracuse (21)	**P7** Fiume Manghisi, Iblei Mts. Syracuse (14)
Trapani (18)	**P8** Riserva Pantalica and Valle Anapo, Iblei Mts. Syracuse (13)
Circum-Sicilian Islands (66)	**P9** Fiume Sosio, Sicani Mts. Agrigento (20)
Aeolian Archipelago (39)	**P10** Riserva Zangara, Belice Valley Trapani (7)
Pelagie Islands (3)	
Pantelleria Island (18)	
Ustica Island (6)	

In bold the wild populations code.

### DNA Extraction and SSR Analysis

Total genomic DNA was extracted from young leaves or inner wood of young cuttings. Tissues were ground into fine powder with liquid nitrogen and stored at −80°C until use. The extraction was carried out following the CTAB method (Doyle and Doyle, 1987). DNA was diluted in water to a final concentration of 10 ng/µl, and its quality assessed by spectrophotometric measurements.

Samples were analyzed at 23 SSR loci (Simple Sequence Repeat), i.e., VVS2 (Thomas and Scott, 1993), VVMD5, VVMD6, VVMD7, VVMD17, VVMD21, VVMD24, VVMD25, VVMD27, VVMD28, VVMD32 (Bowers et al., 1996; Bowers et al., 1999), VrZAG62, VrZAG79 (Sefc et al., 1999), VMC1b11 (Zyprian and Töpfer, 2005), VMC4f3.1 (Di Gaspero et al., 2000), VVIb01, VVIh54, VVIn16, VVIn73, VVIp31, VVIp60, VVIq52, and VVIv67 (Merdinoglu et al., 2005). Forward primers were labeled with one of four fluorescent dyes: 6-FAM, ATTO550, ATTO565, or Yakima Yellow. SSRs were grouped in six multiplex pools, each comprising three or four SSRs marked by different dyes, and characterized by similar annealing temperatures (**Supplementary Table S3**). Twenty-ng DNA per sample were amplified in 96 wells plates by using either the MyTaq HS (Bioline) or the DreamTaq HS (ThermoFisher) DNA polymerases with the following conditions: 15 min at 95°C (Taq activation step), followed by seven cycles consisting of 30 s at 94°C (denaturation), 90 s at the appropriate annealing temperature (**Supplementary Table S3**; touch-down step, with temperature decreasing by 1°C each cycle), 1 min at 72°C (extension). Additional 25 cycles with the same conditions maintained the final annealing temperature constant (**Supplementary Table S3**). Finally, the final PCR step was set for 30 min at 60°C. PCR products were size-separated by capillary electrophoresis performed on a genetic analyzer (ABI Data analysis Prism3130, Applied Biosystems, Inc.) by an external service (Eurofins Genomics, Germany). Electropherograms were visually verified by Gene Mapper v. 5.0 software. Allele size was estimated by comparing the fragment peaks with the internal size standard, using the default method for size calling with SSR and the expected repeat size. To correct for amplification shifts among different PCRs, SSR profiles were normalized by including in each amplification run the DNA of standard cultivars Pinot Noir, Sauvignon Blanc, and Zibibbo. For comparison with the published dataset from Riaz et al. (2018), we used a subset of common core of 17 SSRs, excluding VrZAG62, VrZAG69, VVS2, VVMD5, VVMD17, and VVMD6 from our profiles. For normalization among datasets, we used two common varieties, namely Sangiovese (present with the synonym Minutidda in our dataset) and Zibibbo.

### Data Analysis

To identify ferals, i.e., wild accessions with at least one cultivated parent, we performed a parentage analysis between Sicilian *sylvestris* and cultivated accessions using Bayes' theorem with the R/Solomon package (Christie et al., 2013). The identified ferals (2) were removed from the set of genotypes on which the analyses were performed.

Several diversity parameters were estimated using GenAlEx 6.5 (Peakall and Smouse, 2012): the number of alleles per locus (Na), the number of effective alleles per locus (Ne), the observed (Ho) and expected (He) heterozygosity (Nei, 1978; Nei, 1987), and the fixation index (F). At population level, pairwise Nei's genetic distances and Fst value were calculated. Inbreeding coefficients Fis and Fit were calculated using Arlequin ver. 3.5.2.2 (Excoffier and Lischer, 2010).

The NJ (Neighbor-Joining) phylogenetic tree was designed by using R/ggtree package (Yu et al., 2017) with Nei's distance. The bootstrap analysis was performed based on 1,000 resamplings.

Genetic relationships among the studied genotypes were investigated by Discriminant Analysis of Principal Components (DAPC). DAPC, implemented in the R/adegenet (Jombart, 2008), was performed to infer population subdivision of the analyzed collection, regardless of the geographic origin. The number of principal components (PCs) retained was evaluated using the cross-validation procedure.

Principal coordinates analysis (PCoA) was performed by GenAlEx 6.5 *via* Covariance matrix with data standardization.

To identify the number of genetic groups in the wild populations and to investigate their relationships with domesticated cultivars we used the software STRUCTURE version 2.3.4 (Pritchard et al., 2000) that employs a model-based Bayesian clustering method. The estimate of the most likely number of genetic groups (Ks) was performed following Pritchard and Wen (2003) and the simulation study by Evanno et al. (2005), which proposed an *ad hoc* statistic, DELTA K. For each K, 20 independent runs (100,000 burn-in, 1,000,000 Marchov Chain Monte Carlo) were carried out. All runs were performed with the admixture model. The 20 runs were averaged using the software CLUMPP (CLUster Matching and Permutation Program; Jakobsson and Rosenberg, 2007), and shown in histograms using the program Distruct (Rosenberg, 2004). For the hierarchical analysis, samples showing an ancestry value lower than 0.80 to any cluster were removed. The remaining subsets, one for each cluster, were independently subject to a second round of STRUCTURE analysis, as in Emanuelli et al. (2013), following the procedure described above.

## Results

### Flower Characterization

One of the most obvious traits distinguishing *sativa* vs. *sylvestris* subspecies is the flower structure, since wild grapevine is dioecious whereas flowers of *V. vinifera* subs. *sativa* are usually hermaphroditic. In order to verify that plants collected in putative wild populations showed the dioecious phenotype, we analyzed flower morphology either during collection, or in subsequent years for those plants that had been transferred in the germplasm repository. **Supplementary Table S1** indicates the flower morphology for each plant. As expected, all the 170 cultivated plants had hermaphroditic flowers. Among the wild plants, we could assign a gender only to 122 out of 131 plants (93%), since in nine plants flowers and fruits were not evident at the collection time, and the scions did not survive grafting in the germplasm repository. Among the remainders, 68 plants were clearly females and 54 males, thus allowing us to exclude hermaphrodite plants that are usually considered feral or naturalized forms.

### Genetic Diversity in the Sicilian Germplasm

The Sicilian wild and cultivated germplasm was first screened to identify clones and ferals. The four clones and two ferals identified by parentage analysis were then removed from our dataset.

The genetic profiles of the 170 cultivated and 125 wild accessions at 23 nuclear SSR loci are shown in **Supplementary Table S4**, and their statistics in **Table 2**. The total number of alleles (Na) was 314, with a mean value per locus of 13.7. The marker VVIn73 showed the lowest values of Na, effective alleles (Ne), observed and expected heterozygosity (Ho and He), whereas the marker VVMD28 the highest values, with the exception of Ho, where the maximum was present in VrZag62. The F value ranged from −0.056 (VMC1b11) to 0.403 (VVMD17), with a mean value of 0.093.

**Table 2:** Genetic diversity indices calculated for 295 distinct Sicilian genotypes belonging to *sativa* and *sylvestris* accessions.

Locus	N	Na	Ne	Ho	He	F
VVS2	281	14	5.6	0.829	0.821	−0.011
VVMD5	278	17	7.4	0.662	0.865	0.235
VVMD6	284	11	4.8	0.673	0.793	0.151
VVMD7	288	18	5.3	0.823	0.812	−0.014
VVMD17	287	10	3.2	0.408	0.683	0.403
VVMD21	286	15	2.8	0.570	0.646	0.118
VVMD24	283	9	2.9	0.572	0.654	0.124
VVMD25	278	12	4.6	0.705	0.782	0.098
VVMD27	290	13	6.0	0.807	0.833	0.032
VVMD28	262	24	8.8	0.748	0.887	0.156
VVMD32	276	13	5.7	0.783	0.825	0.052
VrZag62	283	11	7.4	0.859	0.865	0.007
VrZag79	281	13	5.2	0.722	0.809	0.107
VMC1b11	285	16	4.5	0.821	0.778	−0.056
VMC4f3.1	291	17	8.7	0.832	0.885	0.061
VVIb01	280	11	2.9	0.639	0.656	0.026
VVIh54	281	16	3.7	0.541	0.727	0.255
VVIn16	283	8	3.4	0.640	0.704	0.092
VVIn73	295	8	1.4	0.268	0.308	0.131
VVIp31	276	17	7.7	0.793	0.870	0.088
VVIp60	282	15	4.3	0.745	0.769	0.031
VVIq52	284	9	3.5	0.739	0.712	−0.039
VVIv67	284	17	6.5	0.768	0.846	0.092
Mean	282.5	13.7	5.06	0.693	0.762	0.093
Standard Error	1.348	0.809	0.418	0.030	0.026	0.022
Total		314				

Mean value over total samples for each Locus: N, sample size; Na, Number of alleles per locus; Ne, Number of effective alleles; Ho, Observed heterozygosity; He, Expected heterozygosity; F, Fixation index.

Genetic diversity analysis at population level shows that the number of alleles (Na) was similar between the cultivated pool (11.8) and the wild pool (10.8 as average), with wild populations ranging from 2.8 (P3, P4) to 7.0 (P9) (**Table 3**). For the number of effective alleles (Ne), the lowest value was in P4. In the cultivated pool, the observed and expected heterozygosity (Ho and He) were similar (0.697 and 0.741, respectively). The fixation index (F) and the inbreeding coefficient (Fis) were close to zero (0.067 and 0.025, respectively). In wild populations, P1 showed a marked positive F value (0.143), while in the other populations F was negative or close to zero, with P4 showing the lowest value (−0.567). Similarly Fis was strongly negative in P4 showing the lowest Fis value (−0.715).

**Table 3:** Genetic diversity estimates for wild populations and cultivated grapevines accessions analyzed from Sicily.

Population		N	Na	Ne	Ho	He	F	Fis
*P1 -sylvestris (16)*	Mean	15.1	5.7	3.5	0.578	0.670	0.143	0.076
	SE	0.3	0.4	0.3	0.043	0.026	0.054	0.054
*P2 -sylvestris (14)*	Mean	13.9	5.9	3.8	0.684	0.698	0.024	0.054
	SE	0.1	0.4	0.3	0.039	0.025	0.040	0.039
*P3 - sylvestris (4)*	Mean	3.6	2.8	2.3	0.699	0.527	−0.343	−0.300
	SE	0.1	0.2	0.1	0.056	0.034	0.080	0.092
*P4 - sylvestris (16)*	Mean	15.7	2.8	2.0	0.739	0.452	−0.567	−0.715
	SE	0.2	0.2	0.1	0.080	0.035	0.105	0.107
*P5 - sylvestris (9)*	Mean	8.8	4.2	3.0	0.750	0.632	−0.176	−0.122
	SE	0.1	0.2	0.2	0.047	0.027	0.048	0.049
*P6 - sylvestris (12)*	Mean	12.0	5.7	3.3	0.612	0.653	0.072	0.089
	SE	0.0	0.3	0.3	0.042	0.026	0.044	0.043
*P7 - sylvestris (14)*	Mean	13.6	4.4	3.1	0.760	0.634	−0.200	−0.233
	SE	0.1	0.3	0.2	0.042	0.027	0.046	0.046
*P8 - sylvestris (13)*	Mean	12.5	6.0	3.9	0.699	0.710	0.015	−0.006
	SE	0.2	0.4	0.3	0.034	0.026	0.035	0.035
*P9 - sylvestris (20)*	Mean	19.2	7.0	4.1	0.680	0.691	0.012	−0.037
	SE	0.1	0.6	0.4	0.038	0.035	0.033	0.033
*P10 - sylvestris (7)*	Mean	7.0	3.8	2.9	0.737	0.627	−0.179	−0.120
	SE	0.0	0.2	0.2	0.052	0.024	0.070	0.070
*Total sylvestris (125)*	Mean	121.2	10.8	4.6	0.689	0.748	0.082	0.029
	SE	0.7	0.8	0.3	0.029	0.025	0.021	0.020
*sativa (170)*	Mean	161.3	11.8	4.7	0.697	0.741	0.067	0.025
	SE	1.3	0.6	0.4	0.037	0.028	0.031	0.031
*range*		3.6–161.3	2.7–11.7	1.9–4.6	0.57–0.76	0.45–0.74	−0.56-0.14	−0.71–0.08

Mean value over loci for each population. N, number of samples; Na, number of alleles per population; Ne, number of effective alleles; Ho, Observed heterozygosity; He, Expected heterozygosity; F, Fixation index; Fis, inbreeding coefficient (within individuals relative to the rest of their subpopulation); SE, standard error. Numbers in brackets represent the number of accessions per group.

The pairwise Nei's genetic distances and Fst values for all the wild populations and the cultivated pool is shown in **Table 4**. Nei's genetic distance ranged from 0.926 (P4-P5) to 0.083 (P8-cultivated). Fst values confirmed the pattern, with the highest value 0.324 for the pair P5-P4 and the lowest value 0.025 for cultivated-P8. Comparing the wild accessions altogether with the cultivated pool, Nei's genetic distance was 0.147, Fst 0.042 (p < 0.001), Fis 0.02667 (p < 0.001) and Fit 0.07406 (p < 0.001).

**Table 4:** Estimates of pairwise Fst values (below the diagonal) and Unbiased Nei's genetic distance (above the diagonal) within overall wild and cultivated Sicilian accessions.

	P1 *sylvestris*	P2 *sylvestris*	P3 *sylvestris*	P4 *sylvestris*	P5 *sylvestris*	P6 *sylvestris*	P7 *sylvestris*	P8 *sylvestris*	P9 *sylvestris*	P10 *sylvestris*	*sativa*
P1 *sylvestris*		0.161	0.319	0.596	0.270	0.285	0.298	0.182	0.202	0.326	0.291
P2 *sylvestris*	**0.054**		0.192	0.380	0.423	0.210	0.170	0.112	0.134	0.281	0.225
P3 *sylvestris*	**0.094**	**0.061**		0.592	0.377	0.382	0.393	0.270	0.257	0.397	0.334
P4 *sylvestris*	**0.241**	**0.191**	**0.288**		0.926	0.635	0.325	0.298	0.606	0.582	0.315
P5 *sylvestris*	**0.093**	**0.124**	**0.134**	**0.324**		0.467	0.486	0.452	0.300	0.347	0.575
P6 *sylvestris*	**0.096**	**0.071**	**0.125**	**0.268**	**0.148**		0.377	0.243	0.170	0.415	0.361
P7 *sylvestris*	**0.097**	**0.062**	**0.122**	**0.182**	**0.155**	**0.128**		0.193	0.268	0.309	0.263
P8 *sylvestris*	**0.049**	**0.037**	**0.070**	**0.172**	**0.128**	**0.080**	**0.068**		0.167	0.193	0.083
P9 *sylvestris*	**0.061**	**0.043**	**0.072**	**0.233**	**0.095**	**0.059**	**0.088**	**0.049**		0.221	0.310
P10 *sylvestris*	**0.104**	**0.090**	**0.133**	**0.277**	**0.125**	**0.135**	**0.115**	**0.066**	**0.073**		0.268
sativa	**0.079**	**0.062**	**0.081**	**0.138**	**0.137**	**0.100**	**0.078**	**0.025**	**0.080**	**0.077**	

In bold significant Fst values with p ≤ 0.01 calculated over 999 permutations.

**Figure 2:**
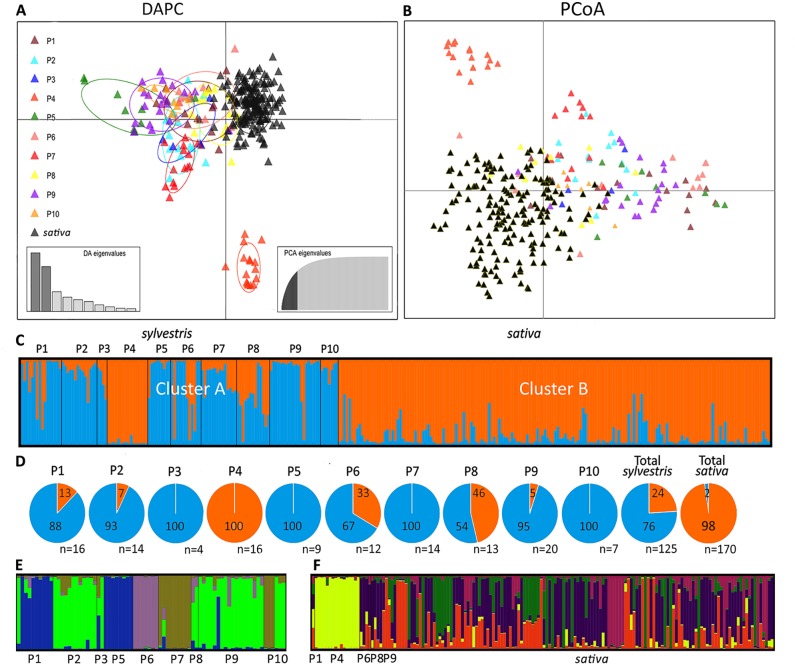
Analyses of Sicilian *sativa* and *sylvestris* germplasm. Discriminant Analysis of Principal Components (DAPC) **(A)**; Principal coordinates analysis (PCoA) **(B)**; first round of STRUCTURE **(C)** with percentage (pies) for each cluster and population **(D)**; second round of STRUCTURE for cluster A **(E)** and cluster B **(F)**.

The genetic diversity of wild and cultivated Sicilian grapevines was first assessed by DAPC analysis of the SSR profiles (**Figure 2A**). The cultivated samples formed a compact cluster in the upper right part of the graph, whereas the wild samples were scattered along the left and the lower sides of the axes. Populations 4 and 5 were the most divergent along the y and x axes, respectively. P4 formed a separate pool, neither related to the other wild populations nor to the cultivated cluster; P5 was less homogeneous and it was clearly connected to other wild populations, yet it stood the furthest apart from the cultivated pool. Interestingly, three samples from wild populations, from P1, P6, and P8, lied amidst the cluster of *sativa*, possibly indicating cases of genetic introgression. Conversely, few cultivated samples fell close to *sylvestris* pools. These cultivars were: Bracaù, Lorisi, Mantonico B, and Tintorè (**Supplementary Dataset S1**).

Samples were also discriminated by PCoA analysis, that is based on genetic distances (**Figure 2B**). The distribution pattern closely resembled the one originated by DAPC, with P4 forming an isolated pool, and the cultivated samples clustering on one side of the main axis. Interestingly, the PCoA confirmed that individual plants from P1, P6, and P8 were admixed within the cultivated cluster.

A third independent analysis of the genetic diversity in Sicilian wild and cultivated germplasm was performed by using STRUCTURE. With this method, the most likely sorting indicated two genetic clusters, A and B (**Figure 2C**, **Supplementary Dataset S1**). Almost all the cultivated plants (98%) belonged to the cluster B (**Figure 2D**). Of these, most of them (95%) had an ancestry value higher than 0.65, and 86% higher than 0.80, indicating a strong link to this cluster (**Supplementary Dataset S1**). Cluster B also included all samples from wild population P4, all of them with ancestry values higher than 0.80, and few individuals from P1, P6, and P8. Cluster A included the majority (76%) of wild plants. Looking in detail the ancestry values of each population, P3, P5, P7, and P10 had all individuals belonging to cluster A. The remaining populations, with the exception of the above mentioned P4, showed variable degrees of association to cluster A (ranging from 95% of P9 to 54% of P8). The association strength to cluster A was high for all wild populations, with ancestry values higher than 0.80 in most cases. The exceptions were P4, as discussed above, and P8, that was equally mixed between the two clusters, and showed low ancestry values (<0.65) in most cases. Interestingly, the cluster A also included four cultivated plants (2% of all cultivated). These varieties are Austina bianca, Bracaù, Giugnatica, and Mantonico B. However, the ancestry values were lower than 0.65 for all these plants, indicating a weak association to the cluster.

Since STRUCTURE did not differentiate among the wild populations, except P4, and between P4 and the cultivated pool, we performed a hierarchical STRUCTURE analysis on the outcome of the first round. In the second round, samples from cluster A split into four subclusters (**Figure 2E**, **Supplementary Dataset S1**). Only two populations, P3 and P10, were equally split between two subclusters. Each remaining population belonged exclusively (P2, P5, P6, P7, P8) or predominantly (90% P1; 94% P9) to single subclusters. For cluster B, the second round of STRUCTURE revealed five subclusters (**Figure 2F**, **Supplementary Dataset S1**). While the cultivated accessions and the few wild individuals of P1, P6, P8, and P9 (that in the first round grouped in cluster B) showed a very mixed pattern among the five subclusters, all individuals from P4 strongly grouped together in a private subcluster (ancestry value > 0.80 in all cases; **Supplementary Dataset S1**).

Finally, genetic distances among the Sicilian samples were also visualized in a phylogenetic tree (**Supplementary Figure S1**). The tree confirmed that most of *sylvestris* populations formed compact branches, indicating that individuals within a population were closely related with each other. The exception was P8 and a small part of P1, P6, and P9, whose individuals were interspersed among the cultivated samples.

### Relationship of Sicilian vs. Mediterranean and Central Asian Germplasm

Recently, Riaz et al. (2018) analyzed a large set of cultivated and wild grapevine accessions from across the Mediterranean basin and Central Asia by 20 nuclear SSRs. In order to frame the genetic structure of the Sicilian germplasm within the geographical distribution of the species, we compared the profiles of 17 SSRs, that represented a common set in the two datasets. The genetic parameters for the markers analyzed are shown in **Table 5**. Overall, ranges and mean values of each parameter were similar to those of the Sicilian germplasm. In the wider survey, we observed a higher number of alleles (Na and Ne), indicating an increased polymorphism in the largest dataset, as expected, especially since Central Asian populations are characterized by high genetic diversity (Riaz et al., 2018).

**Table 5:** Genetic diversity indices calculated for 1,673 genotypes from Europe to Asia belonging to *sativa* and *sylvestris* accessions.

Locus	Na	Ne	Ho	He	F
VVMD7	20	8.531	0.771	0.883	0.127
VVMD21	21	3.350	0.489	0.702	0.303
VVMD24	13	4.304	0.648	0.768	0.156
VVMD25	23	5.342	0.738	0.813	0.092
VVMD27	22	5.823	0.686	0.828	0.172
VVMD28	32	8.850	0.730	0.887	0.177
VVMD32	19	11.006	0.732	0.909	0.195
VMC1b11	24	6.919	0.702	0.855	0.179
VMC4f3.1	32	8.038	0.796	0.876	0.091
VVIb01	20	3.637	0.635	0.725	0.125
VVIh54	25	5.781	0.653	0.827	0.210
VVIn16	14	3.173	0.602	0.685	0.121
VVIn73	15	2.170	0.423	0.539	0.216
VMIp31	26	11.012	0.791	0.909	0.130
VVIp60	20	7.152	0.729	0.860	0.152
VVIq52	13	3.927	0.559	0.745	0.250
VVIv67	27	9.553	0.754	0.895	0.158
Mean	21.529	6.387	0.673	0.806	0.168
Standard Error	1.420	0.675	0.026	0.024	0.013
Total	366				

Na, Number of alleles per locus; Ne, Number of effective alleles; Ho, Observed heterozygosity; He, Expected heterozygosity; F, Fixation index.

Since the dataset by Riaz et al. (2018) contains 289 Italian wild accessions, we first compared our Sicilian wild samples against this subset. PCoA analysis showed that the two pools were clearly separated, and that the Sicilian samples were characterized by higher diversity along the second axis (**Supplementary Figure S2**). Therefore, the Sicilian wild dataset was not redundant with the Italian dataset, and it could be compared with all the other samples.

**Figure 3:**
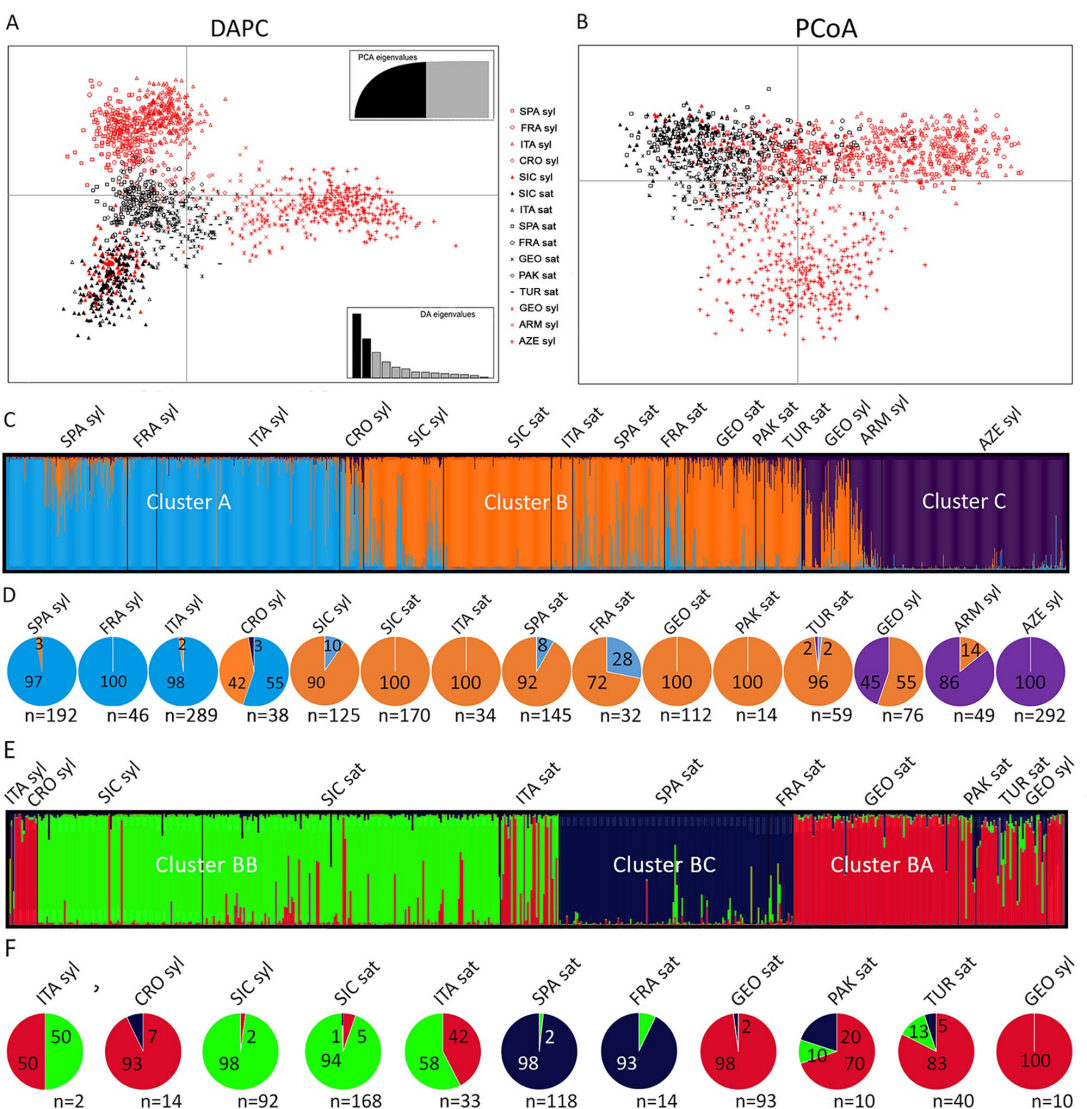
Analyses of Sicilian, Mediterranean and Central Asian *sativa* and *sylvestris* germplasm. Discriminant Analysis of Principal Components (DAPC) **(A)**; Principal coordinates analysis (PCoA) **(B)**; first round of STRUCTURE **(C)** with percentage (pies) for each cluster and population **(D)**; Second round of STRUCTURE for cluster B **(E)** with percentage (pies) for each subcluster and population **(F)**.

The DAPC analysis of all the cultivated and wild accessions from the Mediterranean and Central Asia, including the Sicilian populations, showed a triangle-shaped distribution (**Figure 3A**). The center of the triangle was populated by the wild samples from Croatia and the cultivated accessions from all the regions. The exceptions were Italy and Sicily, which clustered in the lowest vertex, together with all the Sicilian wild populations. The upper vertex included the *sylvestris* samples from Western Countries (Spain, France, Italy), whereas the rightmost vertex included the Eastern *sylvestris* populations (Armenia, Azerbaijan, Georgia). Similarly, the PCoA graph differentiated the Eastern from the Western wild samples. However, all the cultivated samples, including the Sicilian and the Italian, and the wild Sicilian grouped together in this analysis (**Figure 3B**).

We then analyzed all data by STRUCTURE. The addition of the Sicilian germplasm did not vary the most likely number of clusters compared to the original analyses by Riaz et al. (2018), with K = 3 (**Figure 3C**). As in Riaz et al. (2018), one cluster (B) dominated all the cultivated samples, regardless of their origin (**Figure 3D**). A second cluster (C) characterized Asian wild samples. The third cluster (A) was the most abundant for almost all the wild Spanish, French, and Italian samples. The Croatian and Georgian wild samples were especially mixed between cluster A-B and B-C, respectively. A similar situation was observed for the Sicilian wild samples: the major component was cluster B, but many samples showed an important contribution of cluster A. When the major contribution for each sample was considered, 90% of Sicilian wild samples were dominated by cluster B, and 10% by cluster A. Among the 12 samples belonging to cluster A, seven had an ancestry value higher than 0.65, and three exceeded 0.80 (**Supplementary Dataset S2**). When we looked at the distribution within each Sicilian population, only P5 had a large amount of samples (6 out of 9, 67%) belonging to cluster A; six populations (P3, P4, P6, P7, P8, P10) clustered entirely in cluster B; the remaining populations had a few samples in cluster A. All the cultivated Sicilian samples showed cluster B as major component. Nevertheless, Bracaù also had 22% association with cluster A, Austina bianca 19%, and Giugnatica 18%. For all the other samples, the ancestry value for cluster B was higher than 0.80.

Finally, we run a second round of STRUCTURE on the samples closely associated to cluster B, which included most of the Sicilian germplasm and the *sativa* accessions from all the regions. We identified three subclusters (**Figures 3E, F**, **Supplementary Dataset S2**). Subcluster BA included most of the Eastern *sativa* (Georgia 98%; Turkmenistan 83%; Pakistan 70%) and the residual Italian, Croatian, and Georgian *sylvestris* that were grouped in cluster B in the first round. Subcluster BB included most of the Sicilian germplasm, both *sylvestris* (98%) and *sativa* (94%), and more than half of the Italian *sativa* accessions (58%). Subcluster BC included almost all the Spanish and French cultivated accessions (98% and 93%, respectively).

## Discussions

For millennia, grapevine cultivation has been central in the culture and economy of many regions across the Mediterranean Basin and Middle East, with thousands of different varieties selected. Yet, the exact dynamics of grapevine domestication remains elusive, and subject of a passionate debate among scholars of different fields. In the last decades, molecular techniques have expanded our ability to dig into the genetic signatures left along the history of crossing and migrations. Recent studies have generated genetic profiles for hundreds of cultivated and wild grapevine accessions, though the geographical origin of the sampled material is generally unequal, with Eastern and Southern populations highly underrepresented. In this study, we compared the genetic structure of wild and cultivated grapevine germplasm from Sicily, a region that has been so far overlooked. Due to its size and its central position in the Mediterranean, bridging between Europe and Africa, Sicily has always played a key role in the migration routes crossing the Mediterranean basin, both for natural dispersion of species and during human migrations. The recent discovery of a jar containing wine residues dates back the winemaking culture in Sicily to the 4th–3rd millennium BCE, i.e., at approximately the same time of the oldest wine traces found in the Armenia, and about 2,000 years later than the oldest evidence of wine production in Georgia, considered the primary domestication center for grapevine (Areshian et al., 2012; Tanasi et al., 2017; McGovern et al., 2017). For all these reasons, Sicily might have potentially played a major role in the domestication history of grapevine.

We first focused on the comparison between cultivated and wild populations within the Sicilian germplasm. The DAPC and the PCoA distinguished the cultivated from the wild germplasm along the main axis, reflecting major genetic distances between the two groups. Wild samples were especially scattered, indicating a high degree of diversity, probably due to the genetic isolation of most of the populations. The exception was the wild population P4, which separated from the cultivated pool only along the secondary axis, and was not connected with the other wild populations. STRUCTURE confirmed the major divergence between cultivated and wild samples. However, P4 initially grouped together with the cultivated pool, validating the affinity suggested by DAPC and PCoA. Only a second round of STRUCTURE separated P4 from *sativa*, matching the segregation observed along the secondary axis of DAPC and PCoA. These results, together with the Fst, and Nei values, that were highest in the pairwise comparisons of P4 with the other wild groups, suggest that P4 is very different from the other Sicilian wild populations, as confirmed by the Fis value, and that is related to the cultivated accessions. We had already noticed the peculiar genetic makeup of P4 before, though using only six SSR markers (Garfì et al., 2013). It was speculated that the isolation and the unique ecological context of P4 (i.e., a scree-type instead than the usual riparian habitat, **Supplementary Table S2**) likely affected its propagation strategy, relying more on specialized faunal dispersion of seeds than on hydraulic dynamics. In this study, we also observed that P4 was the population showing the highest excess of heterozygosity (F and Fis index), suggesting that it might suffer from genetic introgression from external sources, for example by fertilization of female *sylvestris* plants with pollen from cultivated plants growing nearby, as prompted by the presence of abandoned fields in the surroundings. Intriguingly, all 18 individuals sampled in P4 turned out to be females (**Supplementary Table S1**), a pattern that cannot be explained by random sampling in a natural population of this size. Moreover, we excluded that P4 was a clonal population, spreading by vegetative propagation or apomixy, since genetic analysis revealed that only two plants were clones. All the other individuals were genetically different, though closely related to each other, as evident in the DAPC, PCoA, the second round of STRUCTURE and phylogenetic tree. A possible explanation would be that P4 derives from old dioecious varieties, an uncommon feature in modern cultivated plants, or from hybrids *V. vinifera* x *Vitis* spp., during the early attempts to transfer phylloxera resistance traits to European grapes. That would explain the dioecious phenotype and the affinity with cultivated varieties. We also have to consider that SSRs are neutral molecular markers, whereas the expression of phenotypic traits can be influenced by environmental conditions. Therefore, it is possible that natural settings (forest or riverbank environment, as opposed to agricultural environment) can favor the expression of a more *sylvestris*-like morphology, while more *sativa*-type traits are masked. Finally, during our collection surveys, we deliberately sought plants possessing all the typical *sylvestris* features, such as a dioecious flower, ignoring those with hermaphroditic flowers, that could randomly appear at each generation in some individuals if a population maintains a significant *sativa* contribution. Yet, P4 forms a compact cluster in all the analyses we have performed, therefore the putative introgression events must have occurred in a common ancestor of all current individuals. Alternatively, P4 might represent the residues of an ancient *sylvestris* population that contributed to the genetic structure of many modern Sicilian and Italian cultivars, as discussed below.

Stronger evidence of introgression from the *sativa* pool characterizes P8, since this population largely mixed with the cultivated pool in all our analyses. Moreover, the STRUCTURE ancestry values of most accessions from P8 were weak, indicating mixed profiles between the *sativa* and *sylvestris* clusters. Confirming this hypothesis, the pairwise genetic distance between P8 and the cultivated pool was very low, and individuals from P8 were dispersed among *sativa* accessions in the phylogenetic tree. Population P8 is located in the Anapo Valley at Pantalica, a site with a large Neolithic necropolis that is known to be actively inhabited since ancient times, and that is currently surrounded by cultivated fields. To a minor extent, we also observed evidence of introgression in single individuals of P1 and P6, since a few samples clustered together with the *sativa* group in the DAPC, PCoA, STRUCTURE, and the phylogenetic tree. The genetic isolation of these populations was also confirmed by the positive inbreeding coefficient value (Fis). We suspect introgression with cultivated germplasm also for P3, P5, P7, P10 given that the observed heterozygosity (Ho) values are higher than the expected (He). Nevertheless, the negative Fis value showed by the latter populations, indicating an excess of heterozygosity, could be also due to the following different factors: the small population size, overdominant selection favoring heterozygote survival (heterosis) self-incompatibility system effect, proportion of asexual reproduction and effect of clonal reproduction on the number of heterozygotes (asexuality effect) (Stoeckel et al., 2006). All the other *sylvestris* samples are more isolated, showed different cluster distribution compared to the *sativa* pool and grouped together in the phylogenetic tree, suggesting that their genetic connection with the cultivated pool was weaker. In particular, P5 was the population less related to the *sativa* group, and more similar to the *sylvestris* germplasm from Italy, France, and Spain.

Taking advantage of the extensive study by Riaz et al. (2018), we compared the Sicilian germplasm to cultivated and wild accessions from Western Europe and Central Asia. The dataset used by Riaz et al. (2018) comprises a large number of Italian *sylvestris* (289 accessions). The exact geographical origin of each accession is not specified in the paper, yet we ascertained that Riaz et al.'s dataset does not comprise any Sicilian sample (De Lorenzis, personal communication), as opposed to the larger collection from which the wild Italian samples from Riaz et al. derived (Biagini et al., 2014). Accordingly, we did not find any clone between our dataset and the one from Riaz et al. (2018). Moreover, DAPC and PCoA showed that the Sicilian *sylvestris* samples were very distantly related to the other wild Italian populations, including also those from the neighboring region Calabria, suggesting that the Sicilian wild populations are genetically isolated from the rest of Italy.

By using the combined dataset, the DACP, PCoA, and STRUCTURE analyses confirmed what observed by these authors, with three main clusters discriminating: *i*) a Western *sylvestris* pool, *ii*) an Eastern *sylvestris* pool, and *iii*) the cultivated germplasm, regardless of their origin. The latter cluster also contained the wild Croatian samples. The samples falling in the transition zones among these clusters might suggest events of gene flow between wild populations and the cultivated germplasm in these regions, as previously reported from several investigations (Arroyo-García et al., 2006; Myles et al., 2011; De Andrés et al., 2012; Riaz et al., 2018). In addition to these three main pools, the DAPC also showed a fourth cluster which included most of the Sicilian *sylvestris* plants and all the Sicilian and Italian cultivated samples, suggesting that Sicilian and Italian cultivars are closely related, as expected due to the close geographical proximity, the deep historical connections between the two areas, and the intense commercial exchanges. Moreover, the Sicilian and Italian cultivars were more related with each other, and with the Sicilian *sylvestris*, than with other cultivars worldwide, suggesting events of genetic isolation and/or local secondary domestication, with introgression of genetic material from the Sicilian wild germplasm (possibly, from populations related to current P4) into the cultivated Italian pool. The latter hypothesis is consistent with the assumption that grapevine cultivation in Italy spread from the Southern regions northward since the second part of the 1st millennium BCE (Hopf, 1991; Forni, 2012).

The two-step analysis through STRUCTURE provided some additional information. In the first round of STRUCTURE, the cultivated Sicilian germplasm clustered together with most of the other *sativa* accessions; on the contrary, the wild populations showed a mixed distribution, clustering in part with the cultivated accessions and in part with the Western *sylvestris* pool, a situation similar to what observed in Croatia and, as for the Eastern cluster, in Georgia and Armenia. The different results obtained from STRUCTURE in the analysis of the Sicilian germplasm alone (where the cultivated pool clearly differed from the wild populations) and the wide scale analysis, might depend on the number of SSR markers used (23 in the first analysis and 17 in the second) and on the larger genetic diversity present in the world dataset, that may hinder the smaller differences within the Sicilian accessions. However, the second round of STRUCTURE clearly distinguished a cluster including nearly all the Sicilian germplasm, both *sylvestris* and *sativa*, and more than half of the Italian cultivated accessions, thus confirming the affinity among these groups, already observed in the DAPC analysis. The hierarchical STRUCTURE also separated the cultivated accessions from other regions of the world in two additional clusters. One included almost all samples from Western Europe (Spain and France); a third cluster included the Eastern *sativa* accessions (Georgia, Pakistan, Turkmenistan) plus *sylvestris* form Georgia, Croatia and Italy. Interestingly, about half of the Italian (42%) cultivated accessions also showed affinity for this cluster. Therefore, the Italian cultivars are very different from the rest of Western Europe, and appear as mix between the Eastern group and the Sicilian pool. Accordingly, the list of Italian *sativa* accessions grouping together with the Sicilian germplasm consists predominantly (15 out of 18) of cultivars from Southern regions (Aglianico, Aglianicone, Catarratto Foglia tonda, Frappato, Grillo, Magliocco, Malvasia, Malvasia del Lazio, Malvasia nera di Brindisi, Montonico, Nerello cappuccio, Primitivo, Sangiovese, Sciaccarello, Zibibbo) with the exception of three cultivars that are from Northern Italian regions (Glera, Ribolla gialla, and Schiava lombarda), pointing to a close relationship of this group with the Sicilian germplasm. Conversely, the remaining Italian cultivars, which show affinity with the Eastern pool, are varieties mostly cultivated in the Northern regions (Albarola, Barbera, Brugnola, Butascera, Croatina bianca, Croatina int. corto, Luglienga bianca, Marzemino, Merlina, Moradella di Montalto, Rossara, Rossola, Schiava grossa, Sirica).

The close relationship between the Sicilian *sylvestris* and the Sicilian and Italian *sativa* pools, observed in the wide DAPC, PCoA, and STRUCTURE analyses, is intriguing and can be explained by two different hypotheses. First, it is possible that many Sicilian wild populations suffer from introgression of *sativa* germplasm. This scenario is plausible, considering many different factors, such as the relatively small extension of the island, its millennial history of exploitation, the ancient reduction of its original forest cover, the importance of viticulture in the local economy with extensive fields, and the diffusion of recent diseases threatening the natural populations (Pacifico et al., 2016). We especially found strong evidence for this situation in population P8. Alternatively, it is possible that the current wild Sicilian populations are phylogenetically related to a *sylvestris* group that has not been identified yet, or is even extinct, and that contributed to the early domestication of grapevine. In that case, the residual current Sicilian wild populations maintain a close link to the cultivated germplasm or even directly contributed to the development of some local *sativa* varieties. For its genetic homogeneity, separation from the other *sylvestris* populations and its relation to the cultivated Sicilian and Italian pool, P4 represents an intriguing candidate.

In agreement with this hypothesis, our analyses indicated a few Sicilian cultivated varieties as closely related to the Sicilian wild germplasm, namely Austina bianca, Bracaù, Giugnatica, Lorisi, Mantonico, and Tintorè. Unfortunately, we could retrieve very little historical information for these varieties. Austina bianca is a white grape variety cultivated in the province of Palermo, it was traditionally used for table and wine production. Bracaù, also known as Grecaù, is not mentioned in ancient literature. It is a black berry vine grown in the province of Catania, traditionally used for wine production (Carimi et al., 2010). Giugnatica is a red table grape grown in the Aeolian archipelago. It is considered an early grape that ripens in June. The first citation of Lorisi dates back to the beginning of the nineteenth century. Geremia (1836) mentioned Lorisi, also known as Visparu (Geremia, 1839) in a review of wine varieties found in the vineyards of Etna valley, province of Catania. It was used to make good quality sweet white wines and for the production of raisins. In the second half of the nineteenth century, Caruso (1869) mentions two forms of Lorisi (white and black berry) grown in the area of Cefalù, province of Palermo. Mantonico, existing as white and black berry versions, has been described in Sicily under different names: Muntonicu, Montonico nero femminino, Mantonicu niuru fimmineddu, Montonico nero and Mantonicu niuru for the black berry version, and Montonico bianco, Mantonicu vrancu, Mantonicu masculu for the white berry version. The first citations of this vine dates back to the early 1500s (Venuti, 1516), and later on it was also mentioned by Cupani (1696), Sestini (1812) and Minà Palumbo (1891). In the Aeolian archipelago, where our accession was collected, the red grape form is considered a traditional local variety and is used to produce sweet wines (Gristina et al., 2017). The Tintorè grape, of unknown origin, was found in the province of Agrigento and used to darken the wine. There is no historical information on this grape variety in Sicily. Our results show that Sicilian wild populations are related to the cultivated Sicilian and Italian germplasm, suggesting events of introgression and/or domestication of local varieties. It is thus intriguing to speculate that these ancient Sicilian varieties may derive from local *sylvestris* germplasm.

## Conclusions

The comparison of the genetic structure of Sicilian *sylvestris* populations with the cultivated local germplasm and the grapevine accessions across Western Europe and Central Asia confirms the genetic separation between the Western and Eastern *sylvestris* pools, and their connections with the cultivated germplasm. The Sicilian wild populations appeared closely related to the local cultivated germplasm, probably due to gene flow between the two pools, for either hybridization or early events of introgression of the *sylvestris* germplasm into *sativa* accessions. Considering the archeological evidences that point to Sicily among the oldest centers in grapevine cultivation (Copper Age, early 4th–3rd millennium BCE), it is plausible that the genetic affinity among current Sicilian *sylvestris* and *sativa* germplasms derives from early domestication events occurred in this region. The data set and the results presented here, in a region of primary interest for understanding domestication, migration, and expansion of grape around the Mediterranean basin, may contribute to facilitate future investigations to further unravel the phylogenetic history and population dynamics of grapevine.

## Data Availability Statement

All datasets for this study are included in the article/ **Supplementary Files**.

## Author Contributions

RM and FC conceived and supervised the project. RM, AG, LA, AM, GG and FC contributed to collect plant materials. RM, DC and FB performed the genetic characterization of the plant material. RM, AG and IF analysed the data. RM, AG, IF, and FC analysed and interpreted data. RM, AG, DP and FC wrote the first draft. All authors made a substantial, direct and intellectual contribution to this work. All of the author approve the final version of the manuscript.

## Funding

This research was supported by Regione Siciliana (PSR Sicilia 2007–2013, Sottomisura 214/2A-Preservazione della biodiversità: Centri pubblici di conservazione, Grant No. 94750767637).

## Conflict of Interest

The authors declare that the research was conducted in the absence of any commercial or financial relationships that could be construed as a potential conflict of interest.
