# Supplementary figures and images for: Phylogenetic Relationship Among Wild and Cultivated Grapevine in Sicily: A Hotspot in the Middle of the Mediterranean Basin
Source: Front Plant Sci. 2019 Nov 26;10:1506. doi: 10.3389/fpls.2019.01506 (PMC6888813; doi:10.3389/fpls.2019.01506)

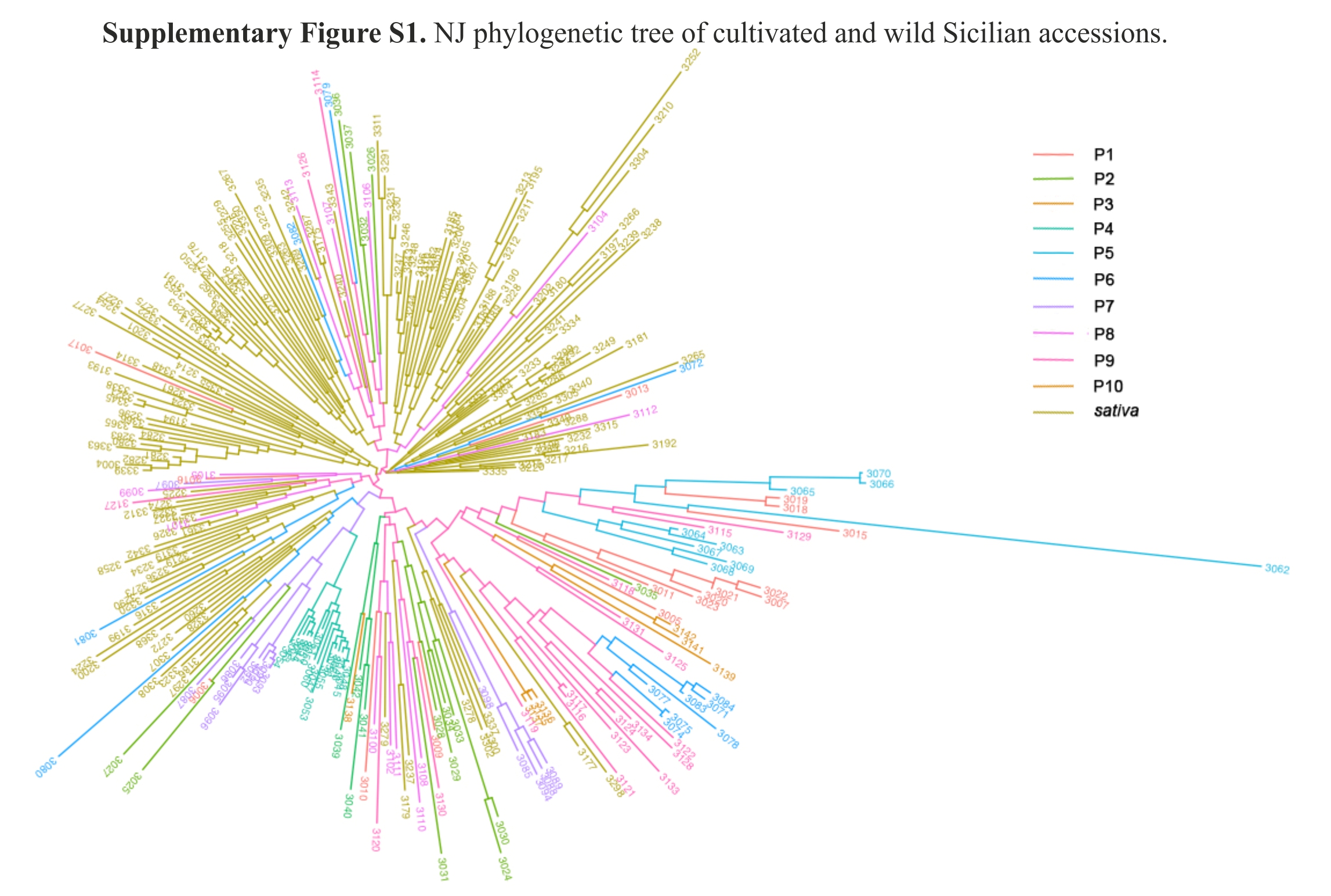

Supplement: Supplementary file 3 [file Image_1.jpg]

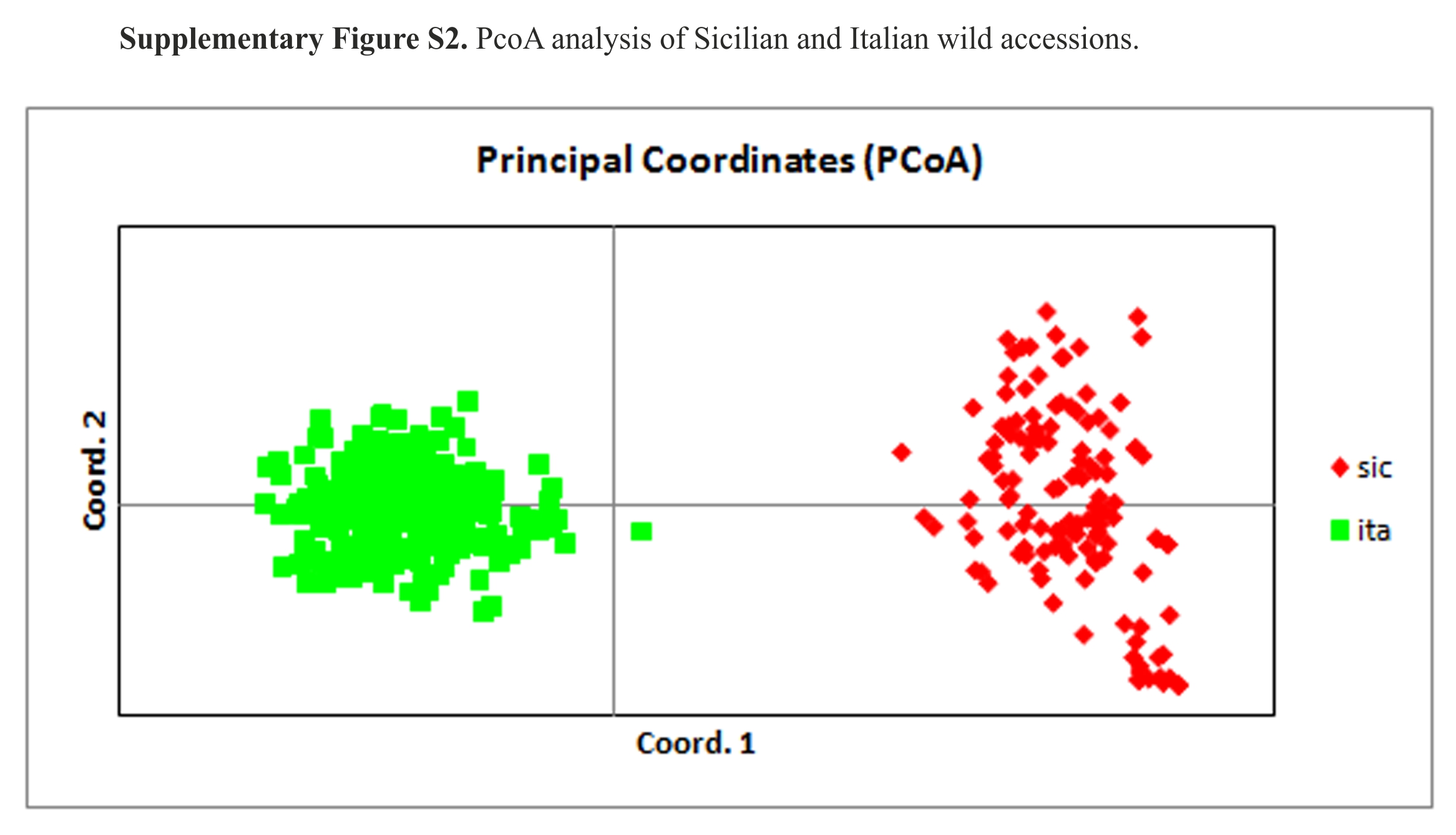

Supplement: Supplementary file 4 [file Image_2.jpg]
