# Supplementary material for: Phylogenetic Relationship Among Wild and Cultivated Grapevine in Sicily: A Hotspot in the Middle of the Mediterranean Basin
Source: Front Plant Sci. 2019 Nov 26;10:1506. doi: 10.3389/fpls.2019.01506 (PMC6888813; doi:10.3389/fpls.2019.01506)
Supplement: Supplementary file 5 [file Table_1.pdf]

Supplementary Table S1. List of 301 *V. vinifera* subps. *sativa* and *sylvestris* grapevine samples analysed.

Accessions ID	Species	Population Code	Source District / Area (Population Code)	Name	Sex
3005	V. vinifera subps. sylvestris	P1	Castelbuono, Madonie Mts. PA (P1)		♀
3006	V. vinifera subps. sylvestris	P1	Castelbuono, Madonie Mts. PA (P1)		♀
3007	V. vinifera subps. sylvestris	P1	Castelbuono, Madonie Mts. PA (P1)		♀
3009	V. vinifera subps. sylvestris	P1	Castelbuono, Madonie Mts. PA (P1)		♀
3010	V. vinifera subps. sylvestris	P1	Castelbuono, Madonie Mts. PA (P1)		♂
3011	V. vinifera subps. sylvestris	P1	Castelbuono, Madonie Mts. PA (P1)		♂
3013	V. vinifera subps. sylvestris	P1	Castelbuono, Madonie Mts. PA (P1)		♀
3015	V. vinifera subps. sylvestris	P1	Castelbuono, Madonie Mts. PA (P1)		♂
3016	V. vinifera subps. sylvestris	P1	Castelbuono, Madonie Mts. PA (P1)		♀
3017	V. vinifera subps. sylvestris	P1	Castelbuono, Madonie Mts. PA (P1)		♀
3018	V. vinifera subps. sylvestris	P1	Castelbuono, Madonie Mts. PA (P1)		♂
3019	V. vinifera subps. sylvestris	P1	Castelbuono, Madonie Mts. PA (P1)		♂
3020	V. vinifera subps. sylvestris	P1	Castelbuono, Madonie Mts. PA (P1)		♀
3021	V. vinifera subps. sylvestris	P1	Castelbuono, Madonie Mts. PA (P1)		♀
3022	V. vinifera subps. sylvestris	P1	Castelbuono, Madonie Mts. PA (P1)		♀
3023	V. vinifera subps. sylvestris	P1	Castelbuono, Madonie Mts. PA (P1)		nd
3024	V. vinifera subps. sylvestris	P2	Cava Grande Cassibile, Iblei Mts. SR (P2)		♂
3025	V. vinifera subps. sylvestris	P2	Cava Grande Cassibile, Iblei Mts. SR (P2)		♂
3026	V. vinifera subps. sylvestris	P2	Cava Grande Cassibile, Iblei Mts. SR (P2)		♀
3027	V. vinifera subps. sylvestris	P2	Cava Grande Cassibile, Iblei Mts. SR (P2)		♂
3028	V. vinifera subps. sylvestris	P2	Cava Grande Cassibile, Iblei Mts. SR (P2)		♀
3029	V. vinifera subps. sylvestris	P2	Cava Grande Cassibile, Iblei Mts. SR (P2)		♂
3030	V. vinifera subps. sylvestris	P2	Cava Grande Cassibile, Iblei Mts. SR (P2)		♂
3031	V. vinifera subps. sylvestris	P2	Cava Grande Cassibile, Iblei Mts. SR (P2)		♀
3032	V. vinifera subps. sylvestris	P2	Cava Grande Cassibile, Iblei Mts. SR (P2)		♀
3033	V. vinifera subps. sylvestris	P2	Cava Grande Cassibile, Iblei Mts. SR (P2)		♀
3034	V. vinifera subps. sylvestris	P2	Cava Grande Cassibile, Iblei Mts. SR (P2)		♂
3035	V. vinifera subps. sylvestris	P2	Cava Grande Cassibile, Iblei Mts. SR (P2)		♀
3036	V. vinifera subps. sylvestris	P2	Cava Grande Cassibile, Iblei Mts. SR (P2)		♂
3037	V. vinifera subps. sylvestris	P2	Cava Grande Cassibile, Iblei Mts. SR (P2)		♀
3039	V. vinifera subps. sylvestris	P3	Cava Sturia, Iblei Mts. SR (P3)		♂
3040	V. vinifera subps. sylvestris	P3	Cava Sturia, Iblei Mts. SR (P3)		♂
3041	V. vinifera subps. sylvestris	P3	Cava Sturia, Iblei Mts. SR (P3)		♀
3042	V. vinifera subps. sylvestris	P3	Cava Sturia, Iblei Mts. SR (P3)		♀
3043	V. vinifera subps. sylvestris	P4	Riserva Boschi Favara e Granza, Torto Valley PA (P4)		♀
3044	V. vinifera subps. sylvestris	P4	Riserva Boschi Favara e Granza, Torto Valley PA (P4)		♀
3045	V. vinifera subps. sylvestris	P4	Riserva Boschi Favara e Granza, Torto Valley PA (P4)		♀
3046	V. vinifera subps. sylvestris	P4	Riserva Boschi Favara e Granza, Torto Valley PA (P4)		♀
3047	V. vinifera subps. sylvestris	P4	Riserva Boschi Favara e Granza, Torto Valley PA (P4)		♀
3048	V. vinifera subps. sylvestris	P4	Riserva Boschi Favara e Granza, Torto Valley PA (P4)		♀
3050	V. vinifera subps. sylvestris	P4	Riserva Boschi Favara e Granza, Torto Valley PA (P4)		♀
3051	V. vinifera subps. sylvestris	P4	Riserva Boschi Favara e Granza, Torto Valley PA (P4)		♀
3052	V. vinifera subps. sylvestris	P4	Riserva Boschi Favara e Granza, Torto Valley PA (P4)		♀
3053	V. vinifera subps. sylvestris	P4	Riserva Boschi Favara e Granza, Torto Valley PA (P4)		♀
3054	V. vinifera subps. sylvestris	P4	Riserva Boschi Favara e Granza, Torto Valley PA (P4)		♀
3055	V. vinifera subps. sylvestris	P4	Riserva Boschi Favara e Granza, Torto Valley PA (P4)		♀
3056	V. vinifera subps. sylvestris	P4	Riserva Boschi Favara e Granza, Torto Valley PA (P4)		♀
3057	V. vinifera subps. sylvestris	P4	Riserva Boschi Favara e Granza, Torto Valley PA (P4)		♀
3058	V. vinifera subps. sylvestris	P4	Riserva Boschi Favara e Granza, Torto Valley PA (P4)		♀
3059	V. vinifera subps. sylvestris	P4	Riserva Boschi Favara e Granza, Torto Valley PA (P4)		♀
3060	V. vinifera subps. sylvestris	P4	Riserva Boschi Favara e Granza, Torto Valley PA (P4)		♀
3061	V. vinifera subps. sylvestris	P4	Riserva Boschi Favara e Granza, Torto Valley PA (P4)		♀
3062	V. vinifera subps. sylvestris	P5	Bosco della Ficuzza, Sicani Mts. PA (P5)		♀
3063	V. vinifera subps. sylvestris	P5	Bosco della Ficuzza, Sicani Mts. PA (P5)		♂
3064	V. vinifera subps. sylvestris	P5	Bosco della Ficuzza, Sicani Mts. PA (P5)		♂
3065	V. vinifera subps. sylvestris	P5	Bosco della Ficuzza, Sicani Mts. PA (P5)		♂
3066	V. vinifera subps. sylvestris	P5	Bosco della Ficuzza, Sicani Mts. PA (P5)		♂
3067	V. vinifera subps. sylvestris	P5	Bosco della Ficuzza, Sicani Mts. PA (P5)		♂
3068	V. vinifera subps. sylvestris	P5	Bosco della Ficuzza, Sicani Mts. PA (P5)		♂
3069	V. vinifera subps. sylvestris	P5	Bosco della Ficuzza, Sicani Mts. PA (P5)		♂
3070	V. vinifera subps. sylvestris	P5	Bosco della Ficuzza, Sicani Mts. PA (P5)		♂
3071	V. vinifera subps. sylvestris	P6	Stretta di Longi, Nebrodi Mts. ME (P6)		♀
3072	V. vinifera subps. sylvestris	P6	Stretta di Longi, Nebrodi Mts. ME (P6)		♀
3074	V. vinifera subps. sylvestris	P6	Stretta di Longi, Nebrodi Mts. ME (P6)		♂
3075	V. vinifera subps. sylvestris	P6	Stretta di Longi, Nebrodi Mts. ME (P6)		♂
3076	V. vinifera subps. sylvestris	P6	Stretta di Longi, Nebrodi Mts. ME (P6)		♂
3077	V. vinifera subps. sylvestris	P6	Stretta di Longi, Nebrodi Mts. ME (P6)		♀
3078	V. vinifera subps. sylvestris	P6	Stretta di Longi, Nebrodi Mts. ME (P6)		♀
3079	V. vinifera subps. sylvestris	P6	Stretta di Longi, Nebrodi Mts. ME (P6)		nd
3080	V. vinifera subps. sylvestris	P6	Stretta di Longi, Nebrodi Mts. ME (P6)		♀
3081	V. vinifera subps. sylvestris	P6	Stretta di Longi, Nebrodi Mts. ME (P6)		♀
3082	V. vinifera subps. sylvestris	P6	Stretta di Longi, Nebrodi Mts. ME (P6)		nd
3083	V. vinifera subps. sylvestris	P6	Stretta di Longi, Nebrodi Mts. ME (P6)		♀
3084	V. vinifera subps. sylvestris	P6	Stretta di Longi, Nebrodi Mts. ME (P6)		♀
3085	V. vinifera subps. sylvestris	P7	Fiume Manghisi, Iblei Mts. SR (P7)		♂
3086	V. vinifera subps. sylvestris	P7	Fiume Manghisi, Iblei Mts. SR (P7)		♂
3087	V. vinifera subps. sylvestris	P7	Fiume Manghisi, Iblei Mts. SR (P7)		♂
3088	V. vinifera subps. sylvestris	P7	Fiume Manghisi, Iblei Mts. SR (P7)		♂

Supplementary Table S1. List of 301 *V. vinifera* subps. *sativa* and *sylvestris* grapevine samples analysed.

Accessions ID	Species	Population Code	Source District / Area (Population Code)	Name	Sex
3089	V. vinifera subps. sylvestris	P7	Fiume Manghisi, Iblei Mts. SR (P7)		♂
3090	V. vinifera subps. sylvestris	P7	Fiume Manghisi, Iblei Mts. SR (P7)		♀
3091	V. vinifera subps. sylvestris	P7	Fiume Manghisi, Iblei Mts. SR (P7)		♀
3092	V. vinifera subps. sylvestris	P7	Fiume Manghisi, Iblei Mts. SR (P7)		♀
3093	V. vinifera subps. sylvestris	P7	Fiume Manghisi, Iblei Mts. SR (P7)		♀
3094	V. vinifera subps. sylvestris	P7	Fiume Manghisi, Iblei Mts. SR (P7)		♂
3095	V. vinifera subps. sylvestris	P7	Fiume Manghisi, Iblei Mts. SR (P7)		♂
3096	V. vinifera subps. sylvestris	P7	Fiume Manghisi, Iblei Mts. SR (P7)		♂
3097	V. vinifera subps. sylvestris	P7	Fiume Manghisi, Iblei Mts. SR (P7)		♀
3098	V. vinifera subps. sylvestris	P7	Fiume Manghisi, Iblei Mts. SR (P7)		♂
3099	V. vinifera subps. sylvestris	P8	Riserva Pantalica e Valle Anapo, Iblei Mts. SR (P8)		♂
3100	V. vinifera subps. sylvestris	P8	Riserva Pantalica e Valle Anapo, Iblei Mts. SR (P8)		♂
3101	V. vinifera subps. sylvestris	P8	Riserva Pantalica e Valle Anapo, Iblei Mts. SR (P8)		nd
3102	V. vinifera subps. sylvestris	P8	Riserva Pantalica e Valle Anapo, Iblei Mts. SR (P8)		nd
3104	V. vinifera subps. sylvestris	P8	Riserva Pantalica e Valle Anapo, Iblei Mts. SR (P8)		♀
3105	V. vinifera subps. sylvestris	P8	Riserva Pantalica e Valle Anapo, Iblei Mts. SR (P8)		♂
3106	V. vinifera subps. sylvestris	P8	Riserva Pantalica e Valle Anapo, Iblei Mts. SR (P8)		♀
3107	V. vinifera subps. sylvestris	P8	Riserva Pantalica e Valle Anapo, Iblei Mts. SR (P8)		♂
3108	V. vinifera subps. sylvestris	P8	Riserva Pantalica e Valle Anapo, Iblei Mts. SR (P8)		♀
3109	V. vinifera subps. sylvestris	P8	Riserva Pantalica e Valle Anapo, Iblei Mts. SR (P8)		♀
3110	V. vinifera subps. sylvestris	P8	Riserva Pantalica e Valle Anapo, Iblei Mts. SR (P8)		♀
3111	V. vinifera subps. sylvestris	P8	Riserva Pantalica e Valle Anapo, Iblei Mts. SR (P8)		♂
3112	V. vinifera subps. sylvestris	P8	Riserva Pantalica e Valle Anapo, Iblei Mts. SR (P8)		♀
3113	V. vinifera subps. sylvestris	P8	Riserva Pantalica e Valle Anapo, Iblei Mts. SR (P8)		♀
3114	V. vinifera subps. sylvestris	P9	Fiume Sosio, Sicani Mts. AG (P9)		♂
3115	V. vinifera subps. sylvestris	P9	Fiume Sosio, Sicani Mts. AG (P9)		♂
3116	V. vinifera subps. sylvestris	P9	Fiume Sosio, Sicani Mts. AG (P9)		♂
3117	V. vinifera subps. sylvestris	P9	Fiume Sosio, Sicani Mts. AG (P9)		♀
3118	V. vinifera subps. sylvestris	P9	Fiume Sosio, Sicani Mts. AG (P9)		♂
3119	V. vinifera subps. sylvestris	P9	Fiume Sosio, Sicani Mts. AG (P9)		♀
3120	V. vinifera subps. sylvestris	P9	Fiume Sosio, Sicani Mts. AG (P9)		♂
3121	V. vinifera subps. sylvestris	P9	Fiume Sosio, Sicani Mts. AG (P9)		♂
3122	V. vinifera subps. sylvestris	P9	Fiume Sosio, Sicani Mts. AG (P9)		♀
3123	V. vinifera subps. sylvestris	P9	Fiume Sosio, Sicani Mts. AG (P9)		♀
3124	V. vinifera subps. sylvestris	P9	Fiume Sosio, Sicani Mts. AG (P9)		♂
3125	V. vinifera subps. sylvestris	P9	Fiume Sosio, Sicani Mts. AG (P9)		♂
3126	V. vinifera subps. sylvestris	P9	Fiume Sosio, Sicani Mts. AG (P9)		nd
3127	V. vinifera subps. sylvestris	P9	Fiume Sosio, Sicani Mts. AG (P9)		♀
3128	V. vinifera subps. sylvestris	P9	Fiume Sosio, Sicani Mts. AG (P9)		♂
3129	V. vinifera subps. sylvestris	P9	Fiume Sosio, Sicani Mts. AG (P9)		♂
3130	V. vinifera subps. sylvestris	P9	Fiume Sosio, Sicani Mts. AG (P9)		♀
3131	V. vinifera subps. sylvestris	P9	Fiume Sosio, Sicani Mts. AG (P9)		♂
3133	V. vinifera subps. sylvestris	P9	Fiume Sosio, Sicani Mts. AG (P9)		♂
3134	V. vinifera subps. sylvestris	P9	Fiume Sosio, Sicani Mts. AG (P9)		♀
3135	V. vinifera subps. sylvestris	P10	Riserva Zangara, Belice Valley TP (P10)		♂
3136	V. vinifera subps. sylvestris	P10	Riserva Zangara, Belice Valley TP (P10)		♂
3137	V. vinifera subps. sylvestris	P10	Riserva Zangara, Belice Valley TP (P10)		nd
3138	V. vinifera subps. sylvestris	P10	Riserva Zangara, Belice Valley TP (P10)		nd
3139	V. vinifera subps. sylvestris	P10	Riserva Zangara, Belice Valley TP (P10)		♀
3140	V. vinifera subps. sylvestris	P10	Riserva Zangara, Belice Valley TP (P10)		♀
3141	V. vinifera subps. sylvestris	P10	Riserva Zangara, Belice Valley TP (P10)		nd
3142	V. vinifera subps. sylvestris	P10	Riserva Zangara, Belice Valley TP (P10)		♂
3143	V. vinifera subps. sylvestris	P10	Riserva Zangara, Belice Valley TP (P10)		♀
3175	V. vinifera subps. sativa	SR	Syracuse (SR)	Albanello	♀♂
3176	V. vinifera subps. sativa	CT	Catania (CT)	Alzano	♀♂
3177	V. vinifera subps. sativa	PA	Palermo (PA)	Austina bianca	♀♂
3178	V. vinifera subps. sativa	PA	Palermo (PA)	Barbarossa	♀♂
3179	V. vinifera subps. sativa	CT	Catania (CT)	Bracaù (Grecaù)	♀♂
3180	V. vinifera subps. sativa	SR	Syracuse (SR)	Bruntisi nero	♀♂
3181	V. vinifera subps. sativa	CT	Catania (CT)	Calabrese	♀♂
3183	V. vinifera subps. sativa	ME	Messina (ME)	Carnuffino	♀♂
3184	V. vinifera subps. sativa	CT	Catania (CT)	Carricante A	♀♂
3185	V. vinifera subps. sativa	CT	Catania (CT)	Carricante C	♀♂
3186	V. vinifera subps. sativa	PA	Palermo (PA)	Catanese nero	♀♂
3187	V. vinifera subps. sativa	TP	Trapani (TP)	Catarratto D	♀♂
3188	V. vinifera subps. sativa	AG	Agrigento (AG)	Catarratto A	♀♂
3189	V. vinifera subps. sativa	TP	Trapani (TP)	Catarratto B	♀♂
3190	V. vinifera subps. sativa	PA	Palermo (PA)	Catarratto C	♀♂
3191	V. vinifera subps. sativa	PA	Palermo (PA)	Catarratto nero	♀♂
3192	V. vinifera subps. sativa	PA	Palermo (PA)	Catarratto Termini	♀♂
3193	V. vinifera subps. sativa	SR	Syracuse (SR)	Cessalà	♀♂
3194	V. vinifera subps. sativa	AG	Agrigento (AG)	Cirrinciò	♀♂
3195	V. vinifera subps. sativa	ME	Messina (ME)	Coda di volpe	♀♂
3196	V. vinifera subps. sativa	EO	Aeolian archipelago (EO)	Corinto A	♀♂
3197	V. vinifera subps. sativa	EO	Aeolian archipelago (EO)	Corinto B	♀♂
3198	V. vinifera subps. sativa	TP	Trapani (TP)	Damaschino A	♀♂
3199	V. vinifera subps. sativa	PA	Palermo (PA)	Diretta bianca	♀♂
3200	V. vinifera subps. sativa	PA	Palermo (PA)	Diretta bianca (Uva di Francia)	♀♂

Supplementary Table S1. List of 301 *V. vinifera* subps. *sativa* and *sylvestris* grapevine samples analysed.

Accessions ID	Species	Population Code	Source District / Area (Population Code)	Name	Sex
3201	V. vinifera subps. sativa	PA	Palermo (PA)	Diretta nera A	♀♂
3202	V. vinifera subps. sativa	CT	Catania (CT)	Dolcetta	♀♂
3203	V. vinifera subps. sativa	RG	Ragusa (RG)	Frappato C	♀♂
3204	V. vinifera subps. sativa	RG	Ragusa (RG)	Frappato 2	♀♂
3205	V. vinifera subps. sativa	RG	Ragusa (RG)	Frappato 3	♀♂
3206	V. vinifera subps. sativa	RG	Ragusa (RG)	Frappato A	♀♂
3207	V. vinifera subps. sativa	RG	Ragusa (RG)	Frappato B	♀♂
3208	V. vinifera subps. sativa	RG	Ragusa (RG)	Frappato F	♀♂
3210	V. vinifera subps. sativa	SR	Syracuse (SR)	Gamay	♀♂
3211	V. vinifera subps. sativa	AG	Agrigento (AG)	Grecanico A	♀♂
3212	V. vinifera subps. sativa	AG	Agrigento (AG)	Grecanico B	♀♂
3213	V. vinifera subps. sativa	TP	Trapani (TP)	Grecanico C	♀♂
3214	V. vinifera subps. sativa	CT	Catania (CT)	Grecaù (Bracaù)	♀♂
3215	V. vinifera subps. sativa	TP	Trapani (TP)	Grillo A	♀♂
3216	V. vinifera subps. sativa	TP	Trapani (TP)	Grillo C	♀♂
3217	V. vinifera subps. sativa	TP	Trapani (TP)	Grillo D	♀♂
3218	V. vinifera subps. sativa	PA	Palermo (PA)	Jala bianca	♀♂
3219	V. vinifera subps. sativa	ME	Messina (ME)	Inzolia A	♀♂
3220	V. vinifera subps. sativa	AG	Agrigento (AG)	Inzolia C	♀♂
3221	V. vinifera subps. sativa	PA	Palermo (PA)	Inzolia imperiale A	♀♂
3223	V. vinifera subps. sativa	CT	Catania (CT)	Inzuccarato	♀♂
3224	V. vinifera subps. sativa	SR	Syracuse (SR)	Inzuccarato di Noto	♀♂
3225	V. vinifera subps. sativa	AG	Agrigento (AG)	Lacrima di Maria A	♀♂
3226	V. vinifera subps. sativa	SR	Syracuse (SR)	Leanfurtisi	♀♂
3227	V. vinifera subps. sativa	PA	Palermo (PA)	Lorisi (Orisi)	♀♂
3228	V. vinifera subps. sativa	ME	Messina (ME)	Lucignola	♀♂
3229	V. vinifera subps. sativa	SR	Syracuse (SR)	Malvagia	♀♂
3230	V. vinifera subps. sativa	EO	Aeolian archipelago (EO)	Malvasia di Lipari C	♀♂
3231	V. vinifera subps. sativa	EO	Aeolian archipelago (EO)	Malvasia di Lipari A	♀♂
3232	V. vinifera subps. sativa	PA	Palermo (PA)	Marsala (Mareschino)	♀♂
3233	V. vinifera subps. sativa	AG	Agrigento (AG)	Marsigliana	♀♂
3234	V. vinifera subps. sativa	PA	Palermo (PA)	Minna di vacca B	♀♂
3235	V. vinifera subps. sativa	ME	Messina (ME)	Minnavacchina	♀♂
3236	V. vinifera subps. sativa	SR	Syracuse (SR)	Minnella bianca (Passulana)	♀♂
3237	V. vinifera subps. sativa	SR	Syracuse (SR)	Monteleone	♀♂
3238	V. vinifera subps. sativa	SR	Syracuse (SR)	Moscato di Noto B	♀♂
3239	V. vinifera subps. sativa	SR	Syracuse (SR)	Moscato bianco	♀♂
3240	V. vinifera subps. sativa	SR	Syracuse (SR)	Moscato di Noto C	♀♂
3241	V. vinifera subps. sativa	SR	Syracuse (SR)	Muscatedda	♀♂
3242	V. vinifera subps. sativa	SR	Syracuse (SR)	Muscatidduni	♀♂
3243	V. vinifera subps. sativa	CT	Catania (CT)	Nerello Mascalese B	♀♂
3244	V. vinifera subps. sativa	CT	Catania (CT)	Nerello Mascalese C	♀♂
3245	V. vinifera subps. sativa	CT	Catania (CT)	Nerello cappuccio A	♀♂
3246	V. vinifera subps. sativa	CT	Catania (CT)	Nerello Mascalese D	♀♂
3247	V. vinifera subps. sativa	CT	Catania (CT)	Nerello Mascalese E	♀♂
3248	V. vinifera subps. sativa	CT	Catania (CT)	Nerello Mascalese A	♀♂
3249	V. vinifera subps. sativa	TP	Trapani (TP)	Nero d'Avola A	♀♂
3250	V. vinifera subps. sativa	PA	Palermo (PA)	Nero grosso	♀♂
3251	V. vinifera subps. sativa	EO	Aeolian archipelago (EO)	Nivureddu	♀♂
3252	V. vinifera subps. sativa	CT	Catania (CT)	Nivuro Bronte	♀♂
3253	V. vinifera subps. sativa	PA	Palermo (PA)	Nucera nera (Perricone)	♀♂
3254	V. vinifera subps. sativa	SR	Syracuse (SR)	Nzuccarato	♀♂
3255	V. vinifera subps. sativa	ME	Messina (ME)	Oriddru	♀♂
3258	V. vinifera subps. sativa	SR	Syracuse (SR)	Osso nivuro	♀♂
3259	V. vinifera subps. sativa	TP	Trapani (TP)	Perricone A	♀♂
3260	V. vinifera subps. sativa	PA	Palermo (PA)	Pizzutella	♀♂
3261	V. vinifera subps. sativa	SR	Syracuse (SR)	Precoce	♀♂
3262	V. vinifera subps. sativa	ME	Messina (ME)	Preventivo	♀♂
3263	V. vinifera subps. sativa	SR	Syracuse (SR)	Racignola bianca	♀♂
3265	V. vinifera subps. sativa	EO	Aeolian archipelago (EO)	Racina di vento	♀♂
3266	V. vinifera subps. sativa	CT	Catania (CT)	Racinedda	♀♂
3267	V. vinifera subps. sativa	SR	Syracuse (SR)	Recunu (Bufania)	♀♂
3269	V. vinifera subps. sativa	EO	Aeolian archipelago (EO)	Rucignola	♀♂
3270	V. vinifera subps. sativa	SR	Syracuse (SR)	Russetto	♀♂
3271	V. vinifera subps. sativa	SR	Syracuse (SR)	Sparo virdisi	♀♂
3272	V. vinifera subps. sativa	PA	Palermo (PA)	Sultanina	♀♂
3273	V. vinifera subps. sativa	PA	Palermo (PA)	Tallone nero	♀♂
3274	V. vinifera subps. sativa	AG	Agrigento (AG)	Tintorè (Ibisu)	♀♂
3275	V. vinifera subps. sativa	PA	Palermo (PA)	Triboti nera	♀♂
3276	V. vinifera subps. sativa	PA	Palermo (PA)	Tripolina bianca	♀♂
3277	V. vinifera subps. sativa	PA	Palermo (PA)	Uva francisi	♀♂
3278	V. vinifera subps. sativa	CT	Catania (CT)	Visparola	♀♂
3279	V. vinifera subps. sativa	ME	Messina (ME)	Vitraruolo	♀♂
3004	V. vinifera subps. sativa	PT	Pantelleria island (PT)	Zibibbo A	♀♂
3280	V. vinifera subps. sativa	PT	Pantelleria island (PT)	Zibibbo augustano	♀♂
3281	V. vinifera subps. sativa	PT	Pantelleria island (PT)	Zibibbo B	♀♂
3282	V. vinifera subps. sativa	PT	Pantelleria island (PT)	Zibibbo carricante	♀♂
3283	V. vinifera subps. sativa	PT	Pantelleria island (PT)	Zibibbo grosso	♀♂

Supplementary Table S1. List of 301 *V. vinifera* subps. *sativa* and *sylvestris* grapevine samples analysed.

Accessions ID	Species	Population Code	Source District / Area (Population Code)	Name	Sex
3284	V. vinifera subps. sativa	PT	Pantelleria island (PT)	Zibibbo nero	♀♂
3285	V. vinifera subps. sativa	TP	Trapani (TP)	Nero d'Avola B	♀♂
3286	V. vinifera subps. sativa	TP	Trapani (TP)	Nero d'Avola C	♀♂
3287	V. vinifera subps. sativa	TP	Trapani (TP)	Nero d'Avola D	♀♂
3292	V. vinifera subps. sativa	TP	Trapani (TP)	Nero d'Avola F	♀♂
3294	V. vinifera subps. sativa	TP	Trapani (TP)	Nero d'Avola G	♀♂
3299	V. vinifera subps. sativa	TP	Trapani (TP)	Nero d'Avola E	♀♂
3304	V. vinifera subps. sativa	CT	Catania (CT)	Alicante, Licante	♀♂
3307	V. vinifera subps. sativa	EO	Aeolian archipelago (EO)	Inzolia imperiale B	♀♂
3308	V. vinifera subps. sativa	EO	Aeolian archipelago (EO)	Cantaro	♀♂
3309	V. vinifera subps. sativa	EO	Aeolian archipelago (EO)	Trummana	♀♂
3310	V. vinifera subps. sativa	EO	Aeolian archipelago (EO)	Minutidda	♀♂
3311	V. vinifera subps. sativa	EO	Aeolian archipelago (EO)	Malvasia B	♀♂
3312	V. vinifera subps. sativa	EO	Aeolian archipelago (EO)	Livedda	♀♂
3313	V. vinifera subps. sativa	EO	Aeolian archipelago (EO)	Mascarisi	♀♂
3314	V. vinifera subps. sativa	EO	Aeolian archipelago (EO)	Racina i mustu B	♀♂
3315	V. vinifera subps. sativa	EO	Aeolian archipelago (EO)	Nuciddara	♀♂
3316	V. vinifera subps. sativa	EO	Aeolian archipelago (EO)	Moscato nero	♀♂
3317	V. vinifera subps. sativa	EO	Aeolian archipelago (EO)	Diretta nera B	♀♂
3319	V. vinifera subps. sativa	EO	Aeolian archipelago (EO)	Minnilottina	♀♂
3320	V. vinifera subps. sativa	EO	Aeolian archipelago (EO)	Mantonico A	♀♂
3321	V. vinifera subps. sativa	EO	Aeolian archipelago (EO)	Cornicchiola A	♀♂
3322	V. vinifera subps. sativa	EO	Aeolian archipelago (EO)	Lacrime di Maria B	♀♂
3323	V. vinifera subps. sativa	EO	Aeolian archipelago (EO)	Inzolia nera A	♀♂
3324	V. vinifera subps. sativa	EO	Aeolian archipelago (EO)	Fiore d'arancio (Trunzu)	♀♂
3325	V. vinifera subps. sativa	EO	Aeolian archipelago (EO)	Lugliatica (Luglienga)	♀♂
3326	V. vinifera subps. sativa	EO	Aeolian archipelago (EO)	Lacrime di Maria C	♀♂
3327	V. vinifera subps. sativa	PT	Pantelleria island (PT)	Greca	♀♂
3328	V. vinifera subps. sativa	PT	Pantelleria island (PT)	Caleu	♀♂
3329	V. vinifera subps. sativa	PT	Pantelleria island (PT)	Minna di vacca C	♀♂
3333	V. vinifera subps. sativa	PE	Pelagie Islands (PE)	Funcia chiatta	♀♂
3335	V. vinifera subps. sativa	PE	Pelagie Islands (PE)	Catarratto E	♀♂
3337	V. vinifera subps. sativa	US	Ustica island (US)	Inzolia nera B	♀♂
3338	V. vinifera subps. sativa	US	Ustica island (US)	Alivedda nera	♀♂
3339	V. vinifera subps. sativa	US	Ustica island (US)	Damaschino B	♀♂
3340	V. vinifera subps. sativa	US	Ustica island (US)	Albanella bianca	♀♂
3342	V. vinifera subps. sativa	US	Ustica island (US)	Menna vacca A	♀♂
3343	V. vinifera subps. sativa	US	Ustica island (US)	Zu Manuele	♀♂
3344	V. vinifera subps. sativa	PT	Pantelleria island (PT)	Centoruotoli	♀♂
3345	V. vinifera subps. sativa	EO	Aeolian archipelago (EO)	Cornicchiola B	♀♂
3348	V. vinifera subps. sativa	RG	Ragusa (RG)	Cutrera	♀♂
3349	V. vinifera subps. sativa	CT	Catania (CT)	Zu Matteo	♀♂
3350	V. vinifera subps. sativa	TP	Trapani (TP)	Catanese bianca	♀♂
3351	V. vinifera subps. sativa	TP	Trapani (TP)	Dunnuni	♀♂
3352	V. vinifera subps. sativa	TP	Trapani (TP)	Fumusa	♀♂
3354	V. vinifera subps. sativa	ME	Messina (ME)	Nocera	♀♂
3355	V. vinifera subps. sativa	EO	Aeolian archipelago (EO)	Prunesta	♀♂
3361	V. vinifera subps. sativa	PT	Pantelleria island (PT)	Inzolia Tunisina A	♀♂
3362	V. vinifera subps. sativa	PT	Pantelleria island (PT)	Nero nostrale	♀♂
3363	V. vinifera subps. sativa	PT	Pantelleria island (PT)	Zibibbo con i seni	♀♂
3364	V. vinifera subps. sativa	PT	Pantelleria island (PT)	Garignano	♀♂
3365	V. vinifera subps. sativa	PT	Pantelleria island (PT)	Zibibbo minna di vacca	♀♂
3366	V. vinifera subps. sativa	PT	Pantelleria island (PT)	Zibibbo masculune	♀♂
3367	V. vinifera subps. sativa	PT	Pantelleria island (PT)	Pionastro	♀♂
3368	V. vinifera subps. sativa	PT	Pantelleria island (PT)	Inzolia Tunisina B	♀♂
3288	V. vinifera subps. sativa	EO	Aeolian archipelago (EO)	Racina i mustu A	♀♂
3289	V. vinifera subps. sativa	EO	Aeolian archipelago (EO)	Cuda i vulpe	♀♂
3290	V. vinifera subps. sativa	EO	Aeolian archipelago (EO)	Pirricone raspo rosso (3°Tipo)	♀♂
3291	V. vinifera subps. sativa	EO	Aeolian archipelago (EO)	Malvasia A	♀♂
3293	V. vinifera subps. sativa	EO	Aeolian archipelago (EO)	Nerello Mascarisi	♀♂
3296	V. vinifera subps. sativa	EO	Aeolian archipelago (EO)	Cappuccio	♀♂
3297	V. vinifera subps. sativa	EO	Aeolian archipelago (EO)	Perricone B	♀♂
3298	V. vinifera subps. sativa	EO	Aeolian archipelago (EO)	Giugnatica	♀♂
3300	V. vinifera subps. sativa	EO	Aeolian archipelago (EO)	Putrisa	♀♂
3302	V. vinifera subps. sativa	EO	Aeolian archipelago (EO)	Mantonico B	♀♂
3305	V. vinifera subps. sativa	EO	Aeolian archipelago (EO)	Mantonico C	♀♂
3334	V. vinifera subps. sativa	PE	Pelagie Islands (PE)	Bertuccio	♀♂

nd: *Sex not determined.*
