# Supplementary material for: Phylogenetic Relationship Among Wild and Cultivated Grapevine in Sicily: A Hotspot in the Middle of the Mediterranean Basin
Source: Front Plant Sci. 2019 Nov 26;10:1506. doi: 10.3389/fpls.2019.01506 (PMC6888813; doi:10.3389/fpls.2019.01506)
Supplement: Supplementary file 6 [file Table_2.pdf]

Supplementary Table S2. Wild grapevine populations analysed: habitat conditions and vegetation patterns.

Population name and Geographical districts	Population code	Habitat	Anthropic evidences	Vegetation patterns and dominant trees/shrubs
Castelbuono, Madonie Mts., Palermo	P1	Temporary river banks	Sporadic drafted, aged olive trees along the track to the sampling area	Riparian forest, canopy cover 100%, h = 5-8 m.<br>*Salix pedicellata, Populusnigra, Laurusnobilis, Quercus ilex, Clematis cirrhosa, Hedera helix, Smilax aspera*
Cava Grande Cassibile, Iblei Mts. Syracuse	P2	Permanent river banks	Small cultivated patches inside the sampling area; Large abandoned and active cultivations in the surroundings	Riparian forest, canopy cover 60%, h = 6-8 m.<br>*Quercus ilex, Fraxinusornus, Salix pedicellata, Ficuscarica, Platanusorientalis, Ailanthus altissima, Pistacialentiscus, Nerium oleander, Erica multiflora, Phlomisfruticosa, Clematis cirrhosa*
Cava Sturia, Iblei Mts. Syracuse	P3	Temporary river banks	Abandoned and active cultivations in the surroundings	Maquis, canopy cover 100%, h = 6-8 m.<br>*Quercus ilex*, *Q. virgiliana, Ficuscarica, Olea europaea*var.*sylvestris, Fraxinusornus, Ailanthus altissima, Pistacialentiscus, P. terebinthus, Rhamnusalaternus, Phillyrealatifolia, Smilax aspera, Hedera helix*
Riserva Boschi Favara and Granza, Torto Valley Palermo	P4	Scree-type deposits	Sporadic small patches of abandoned fields along the track to the sampling area	Pioneer communities, canopy cover 40%, h = 2-4 m.<br>*Ficuscarica, Rubus*sp., *Clematis cirrhosa, Quercusvirgiliana*(along the external border)
Bosco della Ficuzza, Sicani Mts. Palermo	P5	Temporary river banks	Sporadic small patches of abandoned fields and charcoal areas along the stream banks	Riparian forest, canopy cover 100%, h = 15-20 m.<br>*Quercusvirgiliana, Acer campestris, Q. ilex, Fraxinusornus, F. oxyphylla, Q. suber, Salix pedicellata, Populusnigra, Ficuscarica, Crataegusmonogyna, Cytisustriflorus, Rubus, sp., Smilax aspera, Hedera helix, Clematis cirrhosa, Ruscusaculeatus*
Stretta di Longi, Nebrodi Mts. Messina	P6	Permanent river banks	Grazing-disturbance evidences	Riparian forest, canopy cover 100%, h = 8-10 m.<br>*Salix pedicellata, Ficuscarica, Fraxinusangustifolia, Celtisaustralis, Clematis cirrhosa, Hedera helix, Rubus*sp.
Fiume Manghisi, Iblei Mts. Syracuse	P7	Permanent river banks	Abandoned and active cultivations in the surroundings	Riparian forest, canopy cover 80%, h = 8-12 m.<br>*Salix pedicellata, Ficuscarica, Platanusorientalis, Quercus ilex, Clematis cirrhosa, Hedera helix*
Riserva Pantalica and Valle Anapo, Iblei Mts. Syracuse	P8	Permanent river banks	Small cultivated patches inside the sampling area; Large abandoned and active cultivations in the surroundings	Riparian forest, canopy cover 80%, h = 10-15 m.<br>*Salix pedicellata, Populusnigra, Ficuscarica, Platanusorientalis, Quercus ilex, Fraxinusornus, Rhamnusalaternus, Nerium oleander, Clematis cirrhosa*
Fiume Sosio, Sicani Mts. Agrigento	P9	Permanent river banks	Sporadic small patches of abandoned fields along the track to the sampling area	Riparian forest, canopy cover 100%, h = 8-15 m.<br>*Salix pedicellata, Quercus ilex, Q. virgiliana, Crataegusmonogyna, Prunusspinosa, Pistaciaterebinthus, Viburnum tinus, Rhamnusalaternus, Hedera helix*
Riserva Zangara, Belice Valley Trapani	P10	Permanent river banks	Large vineyard in the surroundings. Small abandoned olive patches inside the sampling area	Riparian forest, canopy cover 70%, h = 8-12 m.<br>*Ulmuscanescens, Salix pedicellata, Populusnigra, Ficuscarica, Tamarix*sp.,*Nerium oleander, Myrtuscommunis, Hedera helix*
