# Supplementary material for: Phylogenetic Relationship Among Wild and Cultivated Grapevine in Sicily: A Hotspot in the Middle of the Mediterranean Basin
Source: Front Plant Sci. 2019 Nov 26;10:1506. doi: 10.3389/fpls.2019.01506 (PMC6888813; doi:10.3389/fpls.2019.01506)
Supplement: Supplementary file 7 [file Table_3.pdf]

Supplementary Table S3. SSR marker and PCR multiplex conditions used. The set of six highly reproducible microsatellites suggested by the European group working within the grape GENRES projects is indicated in bold.

SSR marker	Forward primer	Reverse primer	Reference	Core repeat	Annealing T °C (max-min)	Dye	PCR multiplex
**VVS2**	CAG CCC GTA AAT GTA TCC ATC	AAATTCAAAATTCTAATTCAACTGG	Thomas and Scott (1993)	(GA)n	57-50	YAKYE	3
**VVMD5**	CTAGAGCTACGCCAATCCAA	TATACCAAAAATCATATTCCTAAA	Bowers et al. (1996)	(CT)nAT(CT)nATAG(AT)n	62-55	AT565	2
VVMD6	ATCTCTAACCCTAAAACCAT	CTGTGCTAAGACGAAGAAGA	Bowers et al. (1996)	(CT),C(CT),TTAG(CT)TAAT-(CT)C(CT)C(CT)	63-56	AT565	1
**VVMD7**	AGAGTTGCGGAGAACAGGAT	CGAACCTTCACACGCTTGAT	Bowers et al. (1996)	(CT)n	62-55	AT550	2
VVMD17	TGACTCGCCAAAATCTGACG	CACACATATCATCACCACACGG	Bowers et al. (1999)	(CT)n	57-50	AT565	3
VVMD21	GGTTGTCTATGGAGTTGATGTTGC	GCTTCAGTAAAAAGGGATTGCG	Bowers et al. (1999)	(CT)nGAGAAGG(A)n	62-55	YAKYE	4
VVMD24	GTGGATGATGGAGTAGTCACGC	GATTTTAGGTTCATGTTGGTGAAGG	Bowers et al. (1999)	(CT)n	61-54	YAKYE	6
VVMD25	TTCCGTTAAAGCAAAAGAAAAAGG	TTGGATTTGAAATTTATTGAGGGG	Bowers et al. (1999)	(CT)n	57-50	AT550	3
**VVMD27**	GTACCAGATCTGAATACATCCGTAAGT	ACGGGTATAGAGCAAACGGTGT	Bowers et al. (1999)	(CT)n	62-55	FAM	2
VVMD28	AACAATTCAATGAAAAGAGAGAGAGAGA	TCATCAATTTCGTATCTCTATTTGCTG	Bowers et al. (1999)	(CT)n	62-55	YAKYE	2
VVMD32	TATGATTTTTTAGGGGGGTGAGG	GGAAAGATGGGATGACTCGC	Bowers et al. (1999)	(CT)n	63-56	AT550	1
**VrZAG62**	GGTGAAATGGGCACCGAACACACGC	CCATGTCTCTCCTCAGCTTCTCAG	Sefc et al. 1999	(GA)n	63-56	YAKYE	1
**VrZAG79**	AGATTGTGGAGGAGGGAACAAACCG	TGCCCCCATTTTCAAACTCCCTTCC	Sefc et al. 1999	(GA)n	63-56	FAM	1
VMC1b11	CTTTGAAAATTCCTTCCGGGTT	TATTCAAAGCCACCCGTTCTCT	Zyprian and Töpfer (2005)	(GA)n	62-55	FAM	4
VMC4f3.1	AAAGCACTATGGTGGGTGTAAA	TAACCAATACATGCATCAAGGA	Di Gaspero et al. (2000)	(CT)nTT (CT)n	60-53	FAM	5
VVIb01	TGACCCTCGACCTTAAAATCTT	TGGTGAGTGCAATGATAGTAGA	Merdinoglu et al. (2005)	(CT)n	60-53	AT565	5
VVIh54	CCGCACTTGTGTTGAATTTCAG	CAAACCGTTTTTACACCAGCAG	Merdinoglu et al. (2005)	(GA)n	61-54	FAM	6
VVIn16	ACCTCTATAAGATCCTAACCTG	AAGGGAGTGTGACTGATATTTC	Merdinoglu et al. (2005)	(CA)nCG (CA)n	62-55	AT565	4
VVIn73	TACTTCACCTAACAATACAGCT	AATACATAAGGTGAAGATGCCT	Merdinoglu et al. (2005)	(CA)n	60-53	AT550	5
VVIp31	TATCCAAGAGACAAATTCCCAC	TTCTCTTGTTTCCTGCAAATGG	Merdinoglu et al. (2005)	(GA)n	61-54	AT550	6
VVIp60	GGGGAATAACTAAATTGAGGAT	GTATGAATGCGGATAGTTTGTG	Merdinoglu et al. (2005)	(TG)nA(GT)n(GA)n	57-50	FAM	3
VVIq52	TAAAAGGATGGTAGATGACAGA	ACAGGAAAGTGTTCAATGGTTA	Merdinoglu et al. (2005)	(CT)n	62-55	AT550	4
VVIv67	TATAACTTCTCATAGGGTTTCC	TTGGAGTCCATCAAATTCATCT	Merdinoglu et al. (2005)	(CA)nAT(CA)n(GA)nTT(GA)n(AG)n	61-54	AT565	6

SSR markers with the same PCR multiplex number were amplified in a single PCR mix, all primers being pooled in the PCR mix and analyzed in the same sequencer run
