# Supplementary material for: Phylogenetic Relationship Among Wild and Cultivated Grapevine in Sicily: A Hotspot in the Middle of the Mediterranean Basin
Source: Front Plant Sci. 2019 Nov 26;10:1506. doi: 10.3389/fpls.2019.01506 (PMC6888813; doi:10.3389/fpls.2019.01506)
Supplement: Supplementary file 8 [file Table_4.pdf]

Supplementary Table S4. Genetic profiles of cultivated and wild Sicilian accessions.

Accession code	Population / Sample name	VVIp60		VVMD28		VVIb01		VVMD27		VVIv67		VVMD32		VVIn16		VVMD21		VVMD24		VVMD7		VMC1b11		VVIn73		VMIp31		VVIh54		VVIq52		VMC4f3_1		VVMD25		VrZag79		VrZag62		VVMD17		VVMD5		VVMD6		VVS2	
3005	P1	302	302	227	235	290	294	185	185	356	362	0	0	149	151	247	253	206	210	251	253	167	181	257	263	0	0	0	0	80	80	166	178	0	0	243	243	192	192	214	222	231	231	209	209	128	134
3006	P1	314	314	227	243	290	294	179	185	0	0	0	0	151	151	245	245	210	210	243	253	165	167	255	263	182	182	165	165	76	80	182	204	238	254	0	0	0	0	0	0	227	233	209	209	138	138
3007	P1	312	320	235	257	290	290	189	191	356	362	253	255	149	151	253	253	210	212	251	269	181	187	257	263	174	178	163	165	74	74	166	178	238	238	247	247	192	192	214	220	233	233	201	209	128	148
3009	P1	316	316	227	257	288	290	179	179	352	362	251	271	149	151	247	253	206	210	237	243	167	167	263	263	176	186	165	177	74	80	182	202	238	248	245	249	186	198	214	222	233	233	211	211	128	152
3010	P1	314	318	0	0	292	294	181	185	354	358	257	257	151	157	247	255	206	216	263	269	165	187	263	263	182	186	165	165	76	80	170	202	248	262	243	251	192	198	214	224	0	0	201	201	128	130
3011	P1	302	312	235	235	290	290	189	191	352	364	243	255	151	151	247	247	206	210	253	265	165	187	263	263	178	182	149	163	74	74	172	188	240	266	247	247	192	194	222	222	233	233	209	209	128	148
3013	P1	314	314	235	235	294	298	185	189	352	366	251	251	149	149	247	253	206	206	243	253	165	165	263	263	188	188	167	167	74	80	178	188	262	262	247	251	186	200	220	220	233	233	209	211	128	148
3015	P1	302	312	227	249	292	292	181	191	364	364	255	255	149	149	247	247	204	216	253	259	181	187	257	263	182	182	163	163	74	74	166	172	238	238	247	259	194	194	222	222	233	233	201	209	128	148
3016	P1	318	318	227	235	290	290	185	185	0	0	251	251	149	149	247	247	206	210	253	253	165	173	263	263	188	188	163	177	74	80	0	0	248	254	245	249	186	198	214	222	231	231	209	211	128	138
3017	P1	314	318	245	245	290	290	189	193	352	358	239	271	157	157	247	247	210	210	247	253	165	183	261	263	180	192	163	165	76	80	172	172	238	238	251	257	184	186	220	220	227	227	207	207	128	128
3018	P1	312	312	235	235	290	290	189	189	362	364	255	255	149	151	245	245	210	214	253	257	165	181	263	263	180	194	163	163	74	74	172	172	238	248	245	245	194	194	222	222	233	233	201	209	128	148
3019	P1	312	312	235	235	290	290	189	191	362	364	255	255	149	151	245	245	210	214	253	257	181	181	263	263	182	194	163	163	74	80	172	172	238	248	245	245	194	194	222	222	233	233	201	209	128	148
3020	P1	312	320	227	235	290	290	179	189	356	362	0	0	149	149	253	253	210	212	243	251	165	181	257	263	174	178	163	163	74	74	166	178	238	238	0	0	0	0	0	0	233	233	201	201	128	128
3021	P1	312	320	235	257	290	290	189	191	356	362	0	0	149	151	253	253	210	212	251	269	181	187	257	263	174	178	163	165	74	74	166	178	238	238	0	0	0	0	0	0	233	233	201	209	128	148
3022	P1	312	320	235	257	290	290	189	191	356	362	255	255	149	151	253	253	210	212	251	269	181	187	257	263	174	178	163	165	74	74	166	178	238	238	245	245	192	192	214	222	233	233	201	209	128	148
3023	P1	312	312	235	257	290	290	189	191	356	356	255	255	149	151	247	253	206	206	251	269	181	187	257	263	174	188	165	167	74	74	178	206	238	254	247	247	192	192	214	220	227	233	209	209	128	148
3024	P2	302	320	233	271	290	294	185	185	352	366	249	259	147	157	245	245	210	210	237	253	173	181	263	265	182	192	163	163	74	80	202	202	248	254	251	251	194	200	222	222	233	243	209	211	128	134
3025	P2	314	322	227	271	290	294	185	191	362	370	255	271	147	151	245	245	210	210	253	263	165	167	255	265	182	182	165	169	76	80	182	204	238	254	251	251	192	200	214	222	227	233	209	209	138	152
3026	P2	302	318	227	259	290	290	179	189	362	362	251	251	157	157	247	253	206	214	253	257	167	183	257	263	182	186	165	177	74	76	188	188	254	254	247	251	194	198	214	224	227	241	191	209	148	152
3027	P2	314	320	257	257	290	294	181	185	358	362	255	259	151	151	245	253	210	210	257	267	167	187	263	261	182	186	165	165	74	80	172	204	248	248	243	247	198	198	214	224	233	233	201	211	130	138
3028	P2	316	318	227	253	290	290	191	191	352	362	249	255	149	151	247	247	206	210	237	247	165	167	263	263	176	186	165	177	74	80	182	202	238	248	247	247	186	194	222	222	233	233	211	211	128	152
3029	P2	302	318	227	235	288	288	183	185	352	362	251	255	151	151	247	247	206	210	253	253	165	181	263	263	176	182	167	177	74	78	182	188	248	248	251	251	186	200	224	224	233	243	191	211	128	128
3030	P2	302	320	231	241	290	294	185	185	352	352	249	259	147	157	247	247	210	210	237	253	173	181	263	265	182	192	163	163	74	80	202	202	248	248	251	251	194	200	222	222	233	243	207	211	128	134
3031	P2	302	318	227	227	288	294	185	191	356	366	251	255	157	157	241	253	206	210	243	267	169	183	263	265	172	186	149	149	74	80	170	172	262	262	251	251	192	200	222	222	233	233	207	207	128	128
3032	P2	312	314	233	241	290	294	179	189	362	362	251	255	147	151	247	247	0	0	253	253	0	0	263	263	182	186	163	165	74	74	182	170	238	254	247	251	192	198	222	224	227	239	201	201	0	0
3033	P2	302	328	227	235	290	294	185	185	352	366	251	255	149	151	247	247	206	210	247	253	167	181	263	263	182	188	163	177	74	76	172	202	248	248	247	251	186	198	222	222	227	241	211	211	152	152
3034	P2	318	318	225	241	290	290	185	187	352	362	251	255	147	151	247	247	206	210	247	253	167	181	263	263	182	188	163	177	74	78	166	182	248	254	251	251	186	200	214	224	233	243	191	211	128	138
3035	P2	312	314	235	235	294	294	179	189	352	362	239	255	149	151	247	253	210	210	243	269	165	183	263	263	182	182	149	163	72	80	176	178	248	262	247	251	184	192	222	222	227	227	201	209	128	148
3036	P2	302	328	227	227	292	294	179	189	352	362	253	257	149	149	247	255	206	214	257	267	167	181	263	263	182	192	163	163	80	78	170	182	248	254	251	251	192	198	224	224	229	229	207	211	148	152
3037	P2	312	314	235	259	292	294	179	189	362	362	253	257	147	151	247	247	206	210	253	253	181	193	263	263	182	186	163	163	74	74	170	182	238	254	247	251	192	198	224	224	227	241	201	201	138	138
3039	P3	0	0	235	235	288	288	189	189	356	356	251	255	147	149	247	247	206	208	243	253	165	183	263	263	174	188	163	177	0	0	166	204	238	238	251	251	186	192	214	222	229	241	0	0	0	0
3040	P3	312	312	235	257	288	294	185	185	352	362	251	255	147	147	241	247	210	210	253	253	165	183	263	263	174	182	163	163	74	80	174	186	248	262	251	251	198	198	214	222	227	241	191	211	148	152
3041	P3	312	318	233	257	288	288	185	189	352	362	251	255	149	151	241	247	206	210	243	253	165	183	263	263	182	182	165	167	74	80	172	174	240	248	247	251	198	198	214	222	229	241	191	211	148	148
3042	P3	312	318	257	257	0	0	185	189	352	362	0	0	149	151	239	247	206	210	253	255	165	183	263	263	0	0	0	0	74	80	172	174	0	0	243	247	186	198	212	222	239	241	191	211	148	148
3043	P4	314	318	243	257	290	290	181	185	352	358	251	271	149	151	247	247	206	210	253	253	169	183	263	263	186	188	163	175	78	80	182	188	254	254	247	251	198	202	222	222	235	249	209	211	130	138
3044	P4	314	318	243	257	290	290	181	185	352	360	251	271	149	151	247	247	206	210	253	253	169	183	263	263	186	186	165	177	80	80	182	188	254	254	247	251	198	202	222	222	235	249	209	211	130	138
3045	P4	314	318	243	257	290	290	181	185	352	360	253	271	149	151	247	247	206	210	251	253	169	183	263	263	186	186	165	177	80	80	182	188	254	254	247	251	198	202	224	224	235	247	209	211	130	138
3046	P4	314	318	243	257	290	290	181	185	352	360	251	271	149	151	247	247	206	210	251	253	169	183	263	263	186	188	165	177	80	80	182	188	252	254	247	251	198	202	224	224	235	247	209	211	130	138
3047	P4	314	318	243	257	290	290	181	185	352	360	255	263	149	151	247	253	206	210	251	253	169	183	263	263	186	186	165	177	80	78	182	188	254	254	247	251	192	198	214	224	235	247	209	211	130	138
3048	P4	314	318	243	257	290	290	181	185	352	360	251	271	149	151	247	247	206	210	251	253	169	183	263	263	186	186	165	177	80	80	182	188	254	254	247	251	198	202	224	224	235	247	209	211	130	138
3050	P4	0	0	243	257	290	290	181	185	352	358	251	271	149	151	247	247	206	210	251	253	169	183	263	263	186	186	165	177	0	0	182	188	254	254	243	247	198	202	224	224	233	235	0	0	0	0
3051	P4	0	0	235	243	290	290	181	185	352	360	251	271	149	151	247	253	206	210	251	253	169	183	263	263	172	186	165	177	0	0	182	188	254	254	247	251	198	202	224	224	233	235	0	0	0	0
3052	P4	314	318	243	257	290	290	181	185	352	358	251	271	149	151	247	247	206	210	251	253	169	183	263	263	186	186	165	177	80	78	182	188	254	254	247	251	198	202	222	222	235	247	209	211	130	138
3053	P4	314	318	257	257	290	290	181	185	352	354	251	271	149	151	245	245	206	210	243	253	169	183	263	263	186	186	163	177	80	78	182	188	254	254	247	251	198	202	224	224	233	235	209	211	130	138
3054	P4	314	318	243	257	290	290	181	185	352	360	251	271	149	151	247	253	206	210	253	253	169	183	263	263	186	188	165	177	80	78	182	188	252	254	245	249	198	202	222	222	235	249	209	211	130	138
3055	P4	314	318	255	257	290	290	181	185	352	358	251	271	149	151	247	247	206	210	253	253	169	183	263	263	186	186	165	177	80	78	182	188	254	254	247	251	198	202	224	224	235	249	209	211	130	138
3056	P4	314	318	243	257	290	290	181	185	352	358	251	271	149	151	247	247	206	210	253	253	169	183	263	263	186	188	165	177	80	80	182	188	254	254	247	251	198	202	224	224	235	249	209	211	130	138
3057	P4	314	318	243	257	290	290	181	185	352	358	251	271	149	151	247	247	206	210	253	253	169	183	263	263	186	186	165	177	80	80	182	188	254	254	247	251	198	202	222	222	235	249	209	211	130	138
3060	P4	314	318	243	257	290	294	181	185	352	360	251	271	149	151	247	253	206	210	253	251	169	183	263	263	172	172	165	177	80	80	182	188	248	254	247	251	198	202	224	224	235	247	209	211	130	138
3061	P4	314	318	243	257	290	290	181	185	352	358	251	271	149	151	247	247	206	210	253	251	169	183	263	263	186	188	165	177	80	78	182	188	254	254	247	251	198	202	224	224	235	247	209	211	130	138
3062	P5	324	330	231	247	302	302	197	199	344	362	249	263	149	149	217	217	204	204	243	257	181	181	253	253	198	202	141	161	76	76	170	202	238	266	255	255	188	188	222	222	267	267	205	217	132	148
3063	P5	312	316	235	243	288	290	181	191	352	362	239	261	149	149	245	253	208	210	257	261	165	183	263	263	172	194	163	165	76	80	202	202	248	254	247	259	186	192	214	222	235	235	209	209	132	148
3064	P5	0	0	235	243	288	290	181	191	352	362	239	261	149	149	245	253	208	210	257	261	165	183	263	263	172	194	163	163	0	0	202	202	248	254	247	259	186	192	214	222	235	241	0	0	0	0
3065	P5	310	312	235	235	288	290	189	191	354	362	239	255	149	149	247	247	210	214	261	265	165	165	257	263	182	194	163	163	74	76	178	204	248	266	247	247	192	194	212	212	233	241	201	209	128	148
3066	P5	312	312	235	235	288	290	189	191	354	362	239	255	149	149	245	245	210	214	261	265	165	165	257	263	182	194	161	163	74	76	178	202	248	266	247	247	192	194	214	214	233	241	201	207	146	148
3067	P5	312	316	235	243	290	290	181	191	352	362	255	261	149	149	247	253	208	210	253	265	165	183	257	263	172	194	163	163	76	80	172	178	248	254	247	259	186	194	214	222	235	241	201	207	132	148
3068	P5	312	316	235	243	288	294	189	193	352	362	255	259	149	151	247	253	208	210	257	261	165	165	257	263	172	182	163	165	74	80	172	202	248	254	247	257	186	192	214	224	233	235	207	207	132	148
3069	P5	312	316	235	243	288	294	189	189	352	362	255	259	149	151	247	253	208	210	257	259	165	165	257	263	172	182	163	163	74	80	172	204	248	254	247	257	186	192	214	222	233	235	209	209	132	148
3070	P5	312	312	235	235	288	290	189	191	354	362	239	255	149	149	245	245	210	214	261	265	165	165	257	263	182	194	163	163	74	76	178	202	248	266	247	247	192	194	214	214	233	241	201	207	146	148
3071	P6	302	320	245	247	290	294	179	189	354	360	255	269	157	157	247	247	210	210	237	253	165	181	255	263	176	182	163	163	76	74	168	172	238	238	247	251	192	192	214	222	227	235	201	209	152	152
3072	P6	314	322	235	257	290	294	181	195	352	352	255	255	151	151	253	253	204	214	243	243	165	165	263	263	188	188	165	165	80	78	178	182	238	244	251	251	186	186	218	218	235	235	211	211	138	138
3074	P6	312	320	235	247	290	290	189	191	354	358	251	255	157	157	247	247	210	210	265	265	165	165	263	265	180	186	163	163	76	80	172	188	238	238	247	251	192	194	222	222	227	227	201	201	148	152
3075	P6	312	320	235	249	290	290	189	191	354	358	251	255	157	157	247	247	210	210	265	265	165	165	263	265	180	186	163	163	76	80	172	188	238	238	247	251	192	194	222	222	227	227	201	201	148	152
3077	P6	312	320	243	247	290	290	187	189	354	362	251	255	147	157	247	247	210	214	253	265	165	181	263	263	176	186	163	167	76	74	172	172	238	238	247	251	192	194	224	224	227	227	201	201	148	152
3078	P6	312	320	245	249	292	292	189	189	354	362	253	257	147	157	247	247	210	214	253	267	165	181	263	263	176	186	163	167	76	76	172	172	238	238	247	251	192	194	222	222	227	227	201	201	148	152
3079	P6	302	318	227	249	292	294	179	189	352	352	257	257	151	157	241	247	206	210	243	253	165	165	255	255	182	188	163	163	76	80	172	172	238	240	249	259	194	194	224	224	227	227	191	201	138	152
3080	P6	304	314	241	241	298	306	185	219	344	354	271	271	147	149	237	241	206	210	235	251	169	189	253	263	190	204	163	165	76	80	190	182	238	244	251	259	186	212	224	224	227	241	211	211	138	152
3081	P6	302	318	241	241	294	294	179	185	358	366	255	271	155	157	247	253	210	210	237	253	165	165	255	263	178	182	163	165	74	76	168	172	238	254	239	251	184	192	214	224	235	239	207	211	130	152
3082	P6	302	318	233	257	294	294	191	191	352	370	251	251	149	151	247	247	206	206	253	253	183	183	263	263	186	186	165	179	74	80	206	206	240	254	247	247	200	200	224	224	227	241	209	209	138	140
3083	P6	302	320	245	247	290	294	179	189	354	360	255	255	157	157	247	247	210	210	237	253	165	181	255	263	176	182	163	163	76	76	172	178	238	238	247	251	192	194	214	222	227	235	201	209	128	152
3084	P6	302	320	245	247	290	294	179	189	354	360	255	255	157	157	247	247	210	210	237	253	165	181	255	263	176	182	163	163	76	76	168	172	238	238	247	251	192	194	214	222	227	235	201	209	150	152
3085	P7	314	320	257	257	288	290	179	183	370	370	255	263	147	149	247	247	206	214	251	253	181	183	263	265	176	184	163	165	80	80	178	204	248	254	247	251	192	198	214	222	229	241	201	201	128	128
3086	P7	312	318	233	257	290	294	179	191	354	362	0	0	147	151	247	253	206	210	253	265	165	169	263	263	186	190	165	177	80	78	188	206	254	266	247	251	0	0	224	224	233	235	201	201	138	148
3087	P7	312	318	235	235	0	0	179	185	354	360	255	259	149	151	245	253	210	210	243	253	165	183	255	263	0	0	0	0	80	78	172	188	0	0	243	247	198	198	214	222	233	241	201	201	138	148
3088	P7	314	320	227	243	288	290	179	183	370	370	249	249	147	149	247	247	206	214	253	257	181	183	263	265	176	184	165	165	80	78	178	204	248	248	243	247	198	200	222	222	229	241	201	201	128	128
3089	P7	314	320	235	257	288	290	179	183	370	370	249	249	147	149	247	247	206	214	253	257	181	183	263	265	176	184	165	165	80	78	178	204	248	248	247	251	198	200	224	224	229	241	201	201	128	128
3090	P7	312	320	227	233	290	290	185	191	362	370	239	251	149	151	247	253	210	210	243	253	165	183	263	263	176	186	165	177	76	80	188	204	254	266	247	251	198	200	222	222	233	233	201	211	128	138
3091	P7	312	320	227	233	290	290	185	191	362	370	239	251	149	151	247	253	210	210	243	253	165	183	263	263	176	186	165	177	76	80	188	204	254	266	247	251	198	200	222	224	233	249	201	211	128	138
3092	P7	312	320	227	233	290	290	185	191	362	370	239	251	149	151	247	253	210	210	253	253	165	183	263	263	176	186	165	177	76	80	188	204	254	266	247	251	186	192	222	224	233	249	201	211	128	138
3093	P7	312	320	227	233	290	290	185	191	362	370	239	251	149	151	247	253	210	210	253	253	165	183	263	263	176	186	165	177	76	80	188	204	254	266	247	251	198	200	222	224	233	249	201	211	128	138
3094	P7	314	320	245	245	288	290	179	183	370	370	239	249	147	149	247	247	206	214	253	257	181	183	263	265	176	184	165	165	80	78	178	202	248	248	247	251	198	192	214	224	229	241	201	207	128	152

Supplementary Table S4. Genetic profiles of cultivated and wild Sicilian accessions.

Accession code	Population / Sample name	VVIp60		VVMD28		VVIb01		VVMD27		VVIv67		VVMD32		VVIn16		VVMD21		VVMD24		VVMD7		VMC1b11		VVIn73		VMIp31		VVIh54		VVIq52		VMC4f3_1		VVMD25		VrZag79		VrZag62		VVMD17		VVMD5		VVMD6		VVS2	
3095	P7	0	0	233	257	290	294	179	191	354	362	239	255	147	151	253	253	210	210	253	265	165	169	263	263	186	190	165	177	0	0	188	206	254	266	247	251	192	198	232	224	233	235	0	0	0	0
3096	P7	312	318	233	257	290	294	179	191	354	362	239	255	147	151	253	253	210	210	253	265	165	169	263	263	186	190	165	175	78	80	188	206	254	266	247	251	192	198	222	224	233	235	201	201	138	148
3097	P7	318	318	227	235	290	290	185	185	362	370	249	251	149	151	247	253	210	210	243	253	165	183	263	263	176	186	165	165	74	72	202	202	254	254	247	251	192	200	222	222	231	231	207	211	128	128
3098	P7	314	320	235	257	290	290	179	185	352	362	249	271	149	151	247	247	206	206	253	255	181	183	263	265	178	182	165	165	80	78	172	202	248	254	251	251	192	200	214	222	227	227	201	201	128	130
3099	P8	312	312	227	235	290	294	185	185	360	366	251	255	149	151	247	247	210	210	243	269	165	169	263	265	188	188	165	173	74	76	188	206	238	254	239	247	192	192	214	222	231	231	209	211	128	138
3100	P8	312	318	227	235	294	294	185	185	354	354	239	251	151	157	247	247	206	210	243	253	165	169	263	263	186	186	165	173	74	80	182	188	240	248	235	239	192	198	222	222	225	245	209	211	128	152
3101	P8	318	318	227	235	290	306	185	193	366	384	0	0	149	151	247	253	206	210	237	243	165	183	263	263	188	188	167	175	76	80	188	206	248	254	0	0	0	0	0	0	231	231	209	211	132	146
3102	P8	302	318	235	257	294	294	185	191	354	356	251	255	151	151	241	247	206	210	243	253	169	183	263	263	172	186	165	165	74	80	182	206	248	248	243	251	200	200	214	224	241	241	209	209	152	152
3104	P8	316	318	235	243	290	290	185	193	352	362	249	261	151	157	241	253	210	214	243	265	187	193	255	263	174	178	149	163	74	80	188	206	240	240	243	257	186	192	214	214	227	233	209	211	128	140
3105	P8	318	318	227	227	0	0	179	189	362	362	239	255	149	151	247	247	206	210	243	253	165	183	263	263	0	0	0	0	74	76	178	206	0	0	245	257	186	192	222	222	227	233	211	211	138	138
3106	P8	312	318	227	245	290	294	181	191	354	360	249	271	149	157	241	247	206	214	253	259	183	183	263	265	182	186	163	173	74	80	168	188	240	254	247	251	198	198	222	224	227	229	209	209	128	138
3107	P8	314	318	227	235	290	290	183	191	354	362	249	271	151	151	241	247	206	210	251	265	165	181	263	263	174	182	165	165	76	76	188	204	240	254	249	249	192	202	214	224	227	229	209	209	128	138
3108	P8	302	302	227	227	290	290	191	191	352	354	0	0	149	157	247	253	210	210	243	253	165	183	263	263	174	186	173	173	76	80	168	172	238	238	247	251	186	192	212	222	227	233	201	209	128	148
3110	P8	302	318	235	247	292	292	189	191	352	352	251	251	149	179	247	249	206	206	265	267	165	183	261	265	174	186	165	165	74	76	168	172	238	266	247	251	192	194	214	222	233	233	201	209	128	128
3111	P8	302	318	247	247	294	294	185	195	352	356	0	0	147	147	247	247	206	210	243	253	183	183	263	263	172	186	165	165	74	74	188	206	248	248	245	249	0	0	214	224	235	241	201	211	132	152
3112	P8	314	318	243	257	294	298	179	179	354	366	249	251	149	151	245	245	206	210	253	253	169	169	263	263	174	186	167	177	74	80	178	186	240	248	0	0	192	200	212	222	241	241	209	211	128	138
3113	P8	302	300	259	259	290	294	191	195	352	370	251	271	151	151	247	247	206	206	243	243	169	183	263	263	174	182	167	167	76	74	170	170	248	254	247	257	184	194	224	224	227	235	209	211	138	148
3114	P9	302	312	227	259	292	294	189	191	352	366	257	261	147	149	247	257	210	214	237	243	165	165	257	257	174	188	149	163	74	80	172	172	238	254	251	259	192	202	214	222	233	241	207	211	138	148
3115	P9	312	320	227	243	288	290	185	189	362	366	0	0	149	157	247	247	214	214	253	265	165	181	263	263	174	178	149	175	76	80	172	204	248	254	0	0	0	0	214	222	233	233	201	201	148	152
3116	P9	302	314	259	263	290	290	185	185	362	370	241	251	147	149	247	247	206	210	253	267	165	193	263	263	182	194	149	163	74	80	182	206	248	248	251	251	192	194	214	222	223	233	201	211	148	152
3117	P9	302	314	225	257	290	290	185	185	362	370	241	251	147	149	247	247	206	210	253	267	165	193	263	263	182	194	149	163	74	80	182	206	248	248	251	251	192	194	214	222	223	233	201	211	148	152
3118	P9	302	322	227	235	290	294	179	185	352	362	255	255	0	0	247	247	0	0	253	253	165	167	257	263	0	0	163	163	76	80	178	178	0	0	247	251	186	192	214	222	227	241	201	209	138	138
3119	P9	316	318	227	243	288	290	181	191	0	0	257	259	147	149	247	247	206	210	261	265	165	165	263	263	194	194	147	165	74	80	178	178	248	266	247	259	192	194	214	222	0	0	201	209	128	148
3120	P9	312	318	235	263	294	294	185	191	354	354	239	249	147	157	247	255	206	206	253	265	165	181	263	263	186	186	149	163	74	80	172	186	240	248	251	251	192	192	214	214	231	231	201	211	128	152
3121	P9	0	0	257	257	290	290	185	189	356	362	239	255	147	157	247	247	206	210	243	253	165	165	257	257	178	182	149	159	0	0	182	206	248	248	251	259	194	194	214	214	239	241	0	0	0	0
3122	P9	302	312	217	235	288	290	185	189	352	362	249	251	157	157	247	247	210	214	265	265	165	165	263	265	186	188	163	181	76	80	172	204	240	262	251	251	192	192	214	222	227	227	201	201	152	152
3123	P9	314	314	227	263	290	294	189	191	364	366	251	255	149	157	247	247	214	214	251	255	165	165	263	265	174	178	165	175	74	74	178	204	238	252	251	251	192	192	214	222	223	241	201	207	150	152
3124	P9	312	312	227	235	290	290	179	181	362	362	249	249	157	157	247	247	206	214	261	265	165	165	263	263	186	186	165	181	74	74	172	172	238	266	251	259	186	192	214	222	225	231	201	207	128	148
3125	P9	318	322	235	257	294	294	179	181	354	362	249	255	149	157	247	253	206	214	253	265	165	193	263	263	182	182	161	167	74	80	172	188	262	266	257	259	192	198	212	212	233	241	201	211	128	148
3126	P9	318	318	255	255	288	306	179	189	362	370	251	255	149	149	247	247	206	210	243	261	173	179	263	263	188	188	149	173	76	80	178	202	248	254	259	259	192	194	214	224	227	233	209	209	132	152
3127	P9	318	322	243	257	290	290	181	191	352	360	263	263	149	149	247	247	206	208	243	247	165	189	263	263	188	188	163	175	80	80	188	206	248	252	243	247	186	194	222	224	227	239	211	211	130	152
3128	P9	320	328	225	251	290	290	185	189	352	366	249	249	149	157	247	247	210	210	261	265	165	167	257	263	176	176	163	181	74	74	206	206	240	248	251	251	192	192	214	214	227	229	201	207	138	148
3129	P9	312	314	215	227	288	290	181	189	362	364	239	251	149	157	247	247	210	210	265	265	181	181	263	263	172	180	149	149	76	80	172	202	238	266	251	251	192	200	214	214	265	267	201	207	128	148
3130	P9	302	318	241	261	290	294	189	191	362	362	0	0	157	157	247	253	210	210	243	261	165	181	257	263	174	186	149	159	74	80	172	172	238	248	0	0	0	0	0	0	233	239	201	209	0	0
3131	P9	314	318	245	245	290	294	179	191	354	358	255	255	149	157	247	247	214	214	243	253	165	167	263	263	182	182	163	167	74	80	172	172	238	266	247	251	186	192	214	222	233	241	207	211	128	134
3133	P9	302	302	0	0	290	294	181	185	362	362	241	253	147	157	247	247	210	214	243	269	165	165	245	263	182	182	177	181	74	74	160	172	240	266	251	253	192	194	214	224	227	233	201	201	128	152
3134	P9	312	312	227	245	290	290	181	189	362	362	241	249	157	157	247	247	206	214	259	261	165	193	263	263	186	186	165	181	74	74	172	172	238	266	251	259	192	194	214	222	227	235	201	207	128	128
3135	P10	314	318	227	253	290	294	183	189	356	358	241	249	149	151	247	247	214	214	243	253	165	183	263	263	178	182	165	179	74	76	178	178	240	254	247	247	186	192	214	224	241	241	201	209	138	138
3136	P10	0	0	227	253	290	294	183	189	356	358	239	249	149	151	247	247	214	214	243	253	165	183	263	263	178	182	165	179	76	80	178	178	240	254	247	247	186	192	214	224	241	241	201	209	138	138
3137	P10	314	318	227	253	290	294	183	189	356	358	239	249	149	151	247	247	214	214	243	253	165	183	263	263	178	182	165	179	74	76	178	178	240	254	247	247	186	192	214	224	241	241	201	209	138	152
3138	P10	302	312	235	235	288	290	185	189	354	356	255	261	149	149	241	247	204	206	243	253	165	183	263	263	174	188	163	165	74	80	172	202	248	254	251	251	192	194	214	222	227	239	191	207	138	154
3139	P10	312	314	227	233	290	294	181	185	354	362	255	261	149	149	253	253	204	214	243	243	165	167	263	263	174	180	163	177	74	80	202	202	240	248	251	259	192	192	214	214	229	229	191	207	134	154
3141	P10	314	314	227	257	294	294	181	181	356	362	255	255	149	151	247	253	206	214	243	257	267	183	263	265	182	186	165	179	76	76	174	202	248	254	259	259	192	200	214	222	227	233	201	207	128	134
3142	P10	314	314	227	257	294	294	181	185	356	362	255	255	149	151	247	253	206	214	257	265	167	183	263	265	182	186	165	179	76	74	174	202	248	254	259	259	192	200	214	222	227	241	201	207	128	134
3175	Albanello	318	318	243	243	294	306	181	185	356	370	251	271	149	149	247	247	206	206	243	257	165	183	263	263	180	188	165	173	74	76	182	206	254	254	251	259	184	198	224	224	227	229	209	209	132	138
3176	Alzano	318	318	0	0	294	294	179	179	352	360	249	249	149	149	247	247	206	206	243	243	165	183	255	255	176	188	165	165	74	80	166	186	238	238	243	251	186	194	224	224	233	233	207	207	128	142
3177	Austina bianca	314	318	215	267	290	294	185	189	356	358	239	239	157	157	247	263	206	210	243	251	171	173	263	263	180	192	163	167	76	80	170	178	240	254	251	259	192	202	214	214	229	237	201	209	128	138
3178	Barbarossa	318	318	247	253	296	300	183	193	352	360	251	255	149	149	247	253	206	206	243	253	183	183	263	263	182	190	165	165	74	80	188	188	254	268	247	257	186	190	224	224	233	241	191	211	130	144
3179	Bracaù (Grecaù)	302	318	0	0	288	294	189	195	352	370	251	257	147	147	247	253	206	206	251	265	167	167	263	263	176	182	173	173	74	74	172	186	248	248	249	251	186	194	212	222	233	233	191	207	128	138
3180	Bruntisi nero	318	318	235	257	290	290	179	191	0	0	251	271	149	149	247	253	206	206	243	251	165	183	255	255	174	182	163	163	74	80	206	206	240	254	247	257	184	200	222	236	229	229	207	211	128	138
3181	Calabrese	314	314	235	243	300	300	181	195	354	366	251	251	147	147	247	253	206	206	243	243	169	183	263	263	174	188	167	167	80	80	186	186	240	254	251	251	184	200	222	222	225	239	209	211	138	148
3183	Carnuffino	318	318	227	235	290	298	179	191	352	354	251	255	149	149	241	247	206	210	243	253	165	169	263	263	174	186	165	177	80	80	172	172	240	254	243	251	192	200	222	222	229	241	207	209	128	138
3184	Carricante A	314	318	243	247	0	0	179	185	352	352	251	255	149	149	241	241	206	206	239	249	181	181	263	263	0	0	0	0	76	76	166	178	0	0	251	259	192	194	214	224	225	235	191	207	128	128
3185	Carricante C	314	318	243	247	290	290	179	185	352	352	251	251	149	149	241	241	206	206	243	253	181	181	263	263	186	186	163	163	74	74	166	178	238	240	251	259	184	196	212	222	227	237	191	207	128	128
3186	Catanese nero	314	326	233	247	290	306	183	185	352	354	257	271	149	149	241	247	206	206	243	251	165	183	263	263	186	190	165	177	76	80	188	188	238	244	247	249	186	196	224	224	241	241	207	211	128	152
3187	Catarratto D	318	318	227	235	0	0	179	179	354	370	249	251	149	149	241	247	206	206	243	253	165	183	263	265	0	0	0	0	74	80	172	186	0	0	251	251	186	198	224	224	227	227	207	207	128	138
3188	Catarratto A	318	322	227	235	290	298	179	179	354	370	251	271	149	149	241	247	206	206	243	253	165	183	263	263	174	188	163	167	74	74	172	186	254	254	251	251	186	198	224	224	227	233	207	207	128	138
3189	Catarratto B	318	318	227	235	290	298	179	195	352	370	249	249	149	149	241	247	206	206	243	253	165	183	263	263	174	188	163	167	74	80	172	206	254	254	251	251	198	200	222	222	227	233	207	207	128	142
3190	Catarratto C	318	318	233	235	290	298	179	179	354	370	249	251	147	151	241	247	206	206	243	251	169	183	263	265	174	188	163	167	74	80	172	186	254	254	251	251	198	200	222	222	227	227	191	207	128	138
3191	Catarratto nero	318	326	233	257	290	290	183	185	352	352	251	257	155	161	241	247	190	206	243	243	153	163	263	263	190	190	165	165	74	76	190	206	240	254	243	247	186	194	214	224	229	237	191	191	128	138
3192	Catarratto Termini	318	318	241	233	292	300	179	193	352	370	251	271	151	153	247	263	206	208	247	249	181	185	263	263	174	190	165	167	76	76	172	206	246	252	245	249	184	200	222	222	225	227	207	211	138	146
3193	Cessalà	314	326	0	0	290	290	179	195	354	366	271	271	153	153	253	263	206	206	251	257	165	169	263	263	174	184	159	165	74	80	164	202	248	254	251	257	194	202	220	222	0	0	191	211	130	148
3194	Cirrinciò	314	318	233	245	290	294	179	183	352	370	249	251	149	149	241	263	206	214	251	265	165	183	263	263	174	190	165	165	74	74	178	206	240	254	245	251	192	202	214	224	227	237	209	211	128	128
3195	Coda di volpe	322	328	235	235	290	294	179	193	356	370	249	257	147	151	247	247	206	206	243	253	169	183	255	265	174	188	167	167	72	74	186	202	240	254	251	251	198	198	224	224	227	233	201	209	128	138
3196	Corinto A	318	318	0	0	290	294	179	185	352	352	251	251	151	151	0	0	0	0	243	243	165	165	263	263	0	0	165	165	80	80	172	178	240	254	259	259	194	194	212	222	273	273	191	207	128	128
3197	Corinto B	312	314	229	235	288	290	179	189	328	352	251	251	147	149	237	247	204	214	241	243	177	183	263	269	174	180	165	165	76	76	182	206	254	256	247	247	184	196	220	222	227	229	209	209	134	138
3198	Damaschino A	314	318	0	0	0	0	179	195	352	352	249	255	149	151	247	253	0	0	243	243	181	183	263	263	0	0	0	0	80	80	166	166	0	0	251	257	184	186	222	236	227	239	207	207	138	138
3199	Diretta bianca	318	318	233	235	290	294	185	189	334	366	255	271	149	149	253	253	206	210	253	255	167	183	263	265	174	178	147	165	76	78	172	172	240	254	239	259	184	192	222	222	237	239	209	215	128	130
3200	Diretta bianca (Uva di Francia)	306	322	245	245	290	294	189	189	352	366	255	255	151	151	227	247	206	206	253	253	169	187	255	263	190	190	165	165	74	76	168	186	238	254	243	251	200	202	220	222	243	241	201	215	128	138
3201	Diretta nera A	318	318	235	255	0	0	181	189	334	358	239	271	147	151	227	247	204	208	243	249	165	183	253	263	0	0	0	0	76	80	166	178	0	0	251	257	188	202	222	222	225	225	207	207	128	140
3202	Dolcetta	318	318	0	0	290	290	179	195	352	370	251	257	151	151	241	247	206	206	243	243	187	193	255	263	174	182	167	167	74	80	172	206	240	254	243	247	186	194	212	222	225	227	191	207	128	138
3203	Frappato C	318	324	233	233	288	294	179	185	352	358	249	251	149	151	241	247	206	206	243	253	165	187	263	263	186	194	165	173	74	80	178	188	240	254	243	249	192	194	222	222	227	237	191	209	132	138
3204	Frappato 2	318	318	235	257	294	300	179	185	352	360	249	251	149	151	241	247	206	206	243	253	165	187	263	263	186	194	165	173	74	80	178	206	240	254	243	249	192	194	224	224	227	229	207	209	128	138
3205	Frappato 3	314	326	0	0	0	0	179	179	358	358	251	271	149	151	239	241	206	206	243	243	165	187	263	263	0	0	0	0	80	80	178	188	0	0	249	249	192	194	222	222	225	225	191	209	138	146
3206	Frappato A	316	318	227	233	288	294	179	185	352	358	249	249	149	151	241	247	206	206	243	243	165	187	263	263	186	194	165	173	52	64	178	188	240	254	243	249	192	194	222	222	227	227	201	211	130	136
3207	Frappato B	318	318	235	257	288	294	179	185	0	0	249	249	147	151	247	247	206	206	243	243	165	187	263	263	186	194	165	173	80	80	188	178	240	254	243	251	186	198	224	224	225	225	211	211	128	138
3208	Frappato F	318	318	233	257	288	294	179	185	352	358	251	257	149	151	241	247	206	206	243	243	165	187	263	263	186	194	165	173	80	80	178	188	240	254	245	249	186	200	224	224	227	227	211	211	128	138
3210	Gamay	302	318	241	245	288	290	193	193	358	370	249	257	149	157	247	247	204	208	247	251	165	187	255	265	174	174	167	167	74	80	166	186	240	240	251	257	184	186	212	220	227	241	207	207	128	140
3211	Grecanico A	322	328	233	245	290	294	179	193	356	370	249	257	147	149	247	247	206	206	253	257	169	183	255	265	174	188	165	167	74	80	186	202	240	254	251	251	186	198	224	224	225	231	201	207	128	138
3212	Grecanico B	322	328	235	247	0	0	179	193	356	370	249	249	147	149	247	247	206	206	243	253	169	183	255	265	0	0	0	0	74	80	186	202	0	0	251	251	186	198	224	224	227	233	201	209	128	138

Supplementary Table S4. Genetic profiles of cultivated and wild Sicilian accessions.

Accession code	Population / Sample name	VVIp60		VVMD28		VVIb01		VVMD27		VVIv67		VVMD32		VVIn16		VVMD21		VVMD24		VVMD7		VMC1b11		VVIn73		VMIp31		VVIh54		VVIq52		VMC4f3_1		VVMD25		VrZag79		VrZag62		VVMD17		VVMD5		VVMD6		VVS2	
3213	Grecanico C	322	328	235	247	290	294	179	193	356	370	249	257	149	149	247	247	206	206	253	257	169	183	255	263	174	188	167	167	72	72	186	202	240	254	249	249	186	198	224	224	227	233	201	209	128	138
3214	Grecaù (Bracaù)	314	318	227	233	290	294	179	181	360	360	251	255	149	149	247	253	206	208	247	251	165	169	263	263	180	186	163	163	74	80	172	188	236	246	249	253	186	202	222	222	227	227	207	211	128	138
3215	Grillo A	318	318	0	0	290	298	179	195	370	384	251	271	149	155	247	263	206	210	243	253	165	183	263	263	174	190	165	165	74	74	172	180	248	254	247	251	184	200	222	222	225	227	207	211	138	144
3216	Grillo C	318	318	233	239	290	298	179	193	370	384	251	271	149	151	247	263	206	210	247	249	165	183	263	263	174	190	165	167	76	76	172	180	248	254	245	249	184	200	222	222	225	227	207	211	138	144
3217	Grillo D	318	318	233	241	290	298	179	195	370	384	251	271	149	151	247	263	206	210	243	253	165	183	263	263	174	190	165	165	74	80	172	180	248	254	247	251	184	200	220	220	227	227	191	211	138	146
3218	Jala bianca	318	318	267	267	290	294	179	185	352	358	239	249	155	157	241	247	206	206	251	257	169	183	263	263	178	186	165	177	80	80	170	182	238	254	249	255	198	202	224	224	227	233	201	209	134	138
3219	Inzolia A	314	318	243	247	294	306	185	195	370	370	251	257	149	151	247	247	206	206	237	243	179	183	263	263	178	188	165	165	76	80	202	206	238	254	245	249	186	200	222	222	0	0	209	209	132	134
3220	Inzolia C	314	318	227	257	290	298	183	193	352	360	251	257	0	0	0	0	206	206	243	251	0	0	263	263	174	194	165	165	74	76	172	188	240	254	247	249	188	200	222	222	227	227	207	209	130	138
3221	Inzolia imperiale A	314	318	0	0	290	298	179	179	352	366	251	271	149	155	241	247	204	206	0	0	165	183	255	263	184	186	165	165	76	80	166	186	254	254	249	257	184	198	224	224	0	0	207	207	134	138
3223	Inzuccarato	318	326	227	227	0	0	179	193	328	384	257	271	149	149	253	253	206	208	0	0	165	183	263	263	0	0	0	0	74	76	172	188	0	0	241	251	188	188	224	224	225	225	207	207	140	148
3224	Inzuccarato di Noto	318	322	235	245	290	294	185	195	334	366	251	255	149	149	247	253	206	210	253	255	181	183	255	255	178	186	165	165	76	80	166	174	254	254	251	257	184	192	220	220	239	243	209	215	130	130
3225	Lacrima di Maria A	318	318	243	247	0	0	179	193	352	368	249	255	149	149	247	253	206	208	237	243	165	183	263	263	0	0	0	0	78	82	188	206	0	0	249	249	186	198	222	222	227	241	207	211	132	144
3226	Leanfurtisi	314	318	255	255	0	0	179	185	352	370	251	257	151	157	241	247	206	212	241	249	167	171	263	263	0	0	0	0	82	82	172	190	0	0	251	251	188	196	214	224	225	227	191	211	130	146
3227	Lorisi (Orisi)	318	324	235	257	0	0	181	195	362	362	251	257	151	151	241	249	212	212	243	235	165	171	263	263	0	0	165	173	74	76	170	186	238	238	243	251	186	196	214	222	235	243	201	209	132	138
3228	Lucignola	318	322	233	235	290	298	179	179	352	370	249	271	0	0	0	0	0	0	243	243	0	0	263	263	174	190	163	173	74	80	172	172	240	254	241	249	192	200	214	224	227	227	207	207	128	138
3229	Malvagia	318	326	255	255	292	300	179	179	352	352	249	251	151	157	247	253	206	206	241	249	165	169	263	263	174	188	165	165	82	82	172	188	254	254	243	251	188	200	224	224	225	225	191	207	138	146
3230	Malvasia di Lipari C	302	318	233	255	290	294	179	183	348	362	251	271	149	155	247	247	206	214	247	253	165	183	257	263	172	182	157	163	76	80	172	182	240	254	243	247	186	200	222	222	227	227	191	207	138	138
3231	Malvasia di Lipari A	318	326	241	255	290	294	183	183	348	362	251	271	149	155	247	247	206	214	247	253	165	183	257	263	172	182	157	163	76	80	172	182	240	254	243	247	186	200	222	222	227	227	191	207	128	138
3232	Marsala (Mareschino)	314	318	0	0	290	298	179	195	352	366	251	257	151	151	247	253	208	216	243	247	181	183	263	263	174	188	165	165	74	80	166	166	240	254	245	249	186	200	222	222	0	0	191	207	128	140
3233	Marsigliana	314	318	0	0	290	306	183	185	352	356	249	251	149	151	241	247	206	212	243	243	173	183	263	263	188	188	165	165	74	80	172	206	238	254	249	257	184	186	222	222	225	225	191	207	128	128
3234	Minna di vacca B	314	318	249	249	300	310	185	195	370	370	249	251	0	0	0	0	0	0	237	243	0	0	263	263	178	188	163	165	76	80	202	206	238	254	249	255	184	202	222	224	229	239	207	209	132	134
3235	Minnavacchina	318	324	233	255	294	308	191	193	352	370	251	255	149	151	247	247	204	206	241	249	167	183	263	263	182	188	165	175	76	76	206	206	246	252	245	249	186	200	222	224	239	249	209	209	136	138
3236	Minnella bianca (Passulana)	314	318	241	241	294	306	185	195	366	370	249	251	151	153	247	247	206	206	237	243	179	183	263	263	178	188	165	165	76	80	202	206	238	254	251	257	184	202	220	222	229	241	209	211	132	134
3237	Monteleone	302	314	245	245	288	294	189	195	352	370	251	257	149	151	247	247	206	206	251	267	169	181	263	263	176	182	179	173	76	80	172	186	240	248	249	251	192	194	212	224	233	239	191	207	128	138
3238	Moscato di Noto B	314	314	247	267	294	300	179	193	358	370	263	271	147	147	247	263	208	214	237	253	183	187	261	263	182	186	165	165	76	76	166	206	240	248	251	255	184	194	220	222	229	229	207	211	128	138
3239	Moscato bianco	318	324	235	257	290	294	179	193	358	358	263	263	147	147	261	263	206	206	243	253	183	187	261	263	182	188	165	165	74	76	166	206	238	254	255	255	194	194	220	222	241	241	201	209	132	138
3240	Moscato di Noto C	318	318	0	0	290	294	0	0	352	352	251	271	0	0	263	263	0	0	0	0	0	0	263	263	174	188	165	165	74	80	0	0	240	254	249	249	184	200	224	224	0	0	209	209	138	138
3241	Muscatedda	314	318	235	245	290	294	179	179	352	370	257	271	147	157	253	253	206	214	243	253	165	183	263	263	182	182	165	179	74	76	166	182	240	254	247	255	184	186	214	224	229	243	209	209	128	138
3242	Muscatidduni	302	318	257	257	290	294	189	195	352	370	249	251	153	153	247	247	206	206	241	243	169	183	263	263	174	182	167	173	76	76	170	172	248	254	247	257	184	194	224	224	227	235	209	211	138	146
3243	Nerello Mascalese B	314	318	227	241	290	298	179	179	352	352	251	255	149	151	241	247	206	212	243	253	165	169	263	263	172	182	163	163	74	80	172	178	240	240	251	259	194	200	212	222	225	225	191	209	128	138
3244	Nerello Mascalese C	314	318	0	0	290	298	179	179	352	354	251	255	0	0	0	0	0	0	243	253	0	0	263	263	188	194	163	165	80	80	172	178	240	240	251	259	194	200	214	222	225	225	191	209	128	138
3245	Nerello cappuccio A	314	322	247	257	290	294	181	185	352	352	249	251	0	0	0	0	0	0	243	243	0	0	263	263	174	176	165	165	74	76	0	0	240	254	251	259	184	186	222	222	227	229	191	207	128	138
3246	Nerello Mascalese D	314	318	225	241	290	298	179	179	352	354	251	255	151	151	241	247	206	212	243	253	167	171	263	263	188	194	163	163	80	80	172	178	240	240	251	259	194	200	214	222	227	237	191	209	128	138
3247	Nerello Mascalese E	314	318	225	241	290	298	179	179	352	354	251	255	151	153	241	247	206	212	243	253	165	183	263	263	186	188	163	163	74	80	172	178	240	240	251	259	194	200	212	222	227	237	191	209	128	138
3248	Nerello Mascalese A	314	318	241	241	290	298	179	179	352	354	251	255	149	149	241	247	206	206	243	253	165	183	263	263	188	194	163	163	76	82	172	178	240	240	249	257	194	200	212	222	227	239	191	209	128	138
3249	Nero d'Avola A	312	314	235	235	298	298	179	181	354	366	251	251	147	149	247	253	206	210	243	253	169	183	263	263	174	188	165	165	78	80	186	186	240	254	251	251	184	200	220	220	227	227	209	211	138	146
3250	Nero grosso	318	326	231	241	290	290	185	193	352	352	249	271	149	155	241	247	206	210	243	243	167	173	263	263	190	194	165	165	74	78	180	190	248	254	251	255	188	188	224	224	233	233	191	207	130	138
3251	Nivureddu	318	326	233	257	0	0	183	185	352	352	251	257	149	155	241	247	206	206	243	243	165	165	263	263	0	0	165	165	76	76	178	190	0	0	243	247	186	194	214	222	229	237	191	191	128	130
3252	Nivuro Bronte	314	318	0	0	288	290	195	193	358	358	239	239	151	157	241	241	208	208	243	247	187	193	255	263	174	182	167	167	74	80	166	186	240	240	257	257	186	186	212	236	225	225	207	211	138	138
3253	Nucera nera (Perricone)	318	328	233	257	292	292	183	185	352	352	251	257	149	155	241	247	206	206	243	243	165	165	263	263	190	190	165	165	74	76	178	190	240	254	243	247	186	194	212	222	229	237	191	191	128	130
3254	Nzuccarato	318	326	241	255	290	290	179	195	328	328	251	271	151	151	257	257	206	210	247	255	165	165	263	263	190	194	165	175	74	76	172	188	240	244	243	243	186	186	222	222	227	235	207	207	140	150
3255	Oriddru	318	322	0	0	294	298	0	0	354	384	257	259	149	149	241	247	206	206	0	0	169	183	263	263	174	178	165	167	72	76	186	202	246	252	241	245	202	202	224	228	0	0	207	211	128	138
3258	Osso nivuro	302	318	257	257	294	294	189	195	0	0	251	257	153	153	247	255	206	208	237	243	183	187	263	263	186	188	151	165	74	76	168	206	238	238	251	257	186	202	222	222	233	239	191	211	128	134
3259	Perricone A	318	326	233	257	290	290	179	185	352	352	249	257	151	155	241	247	206	206	243	243	165	167	263	263	190	190	165	165	76	80	172	178	240	254	243	247	186	194	214	224	227	229	191	191	128	128
3260	Pizzutella	312	314	257	257	290	294	183	185	352	356	257	271	151	151	253	253	206	206	243	253	183	183	263	263	172	182	165	177	74	76	166	188	248	254	243	251	184	186	222	222	227	233	209	209	128	130
3261	Precoce	314	318	233	245	290	306	189	193	352	360	239	271	0	0	0	0	0	0	247	253	0	0	263	263	180	192	163	165	76	82	172	186	238	238	251	257	184	186	222	224	225	237	209	209	128	128
3262	Preventivo	318	318	233	233	288	290	179	185	352	352	251	251	0	0	0	0	0	0	245	267	0	0	263	263	190	194	165	173	74	80	172	178	240	240	243	251	186	186	214	224	227	239	191	209	128	128
3263	Racignola bianca	318	324	235	257	294	306	179	193	352	352	251	255	151	153	247	247	206	206	243	253	167	183	263	263	184	188	175	179	76	76	172	206	248	254	247	249	188	200	222	224	227	233	209	209	136	138
3265	Racina di vento	314	322	235	257	296	300	181	193	352	352	255	271	149	149	253	253	202	212	243	253	165	187	263	263	188	188	165	165	74	80	178	178	240	246	249	249	188	202	220	224	235	241	209	209	136	138
3266	Racinedda	312	314	0	0	288	290	179	189	328	360	251	251	147	149	237	247	206	214	0	0	177	183	261	269	174	180	165	165	76	76	182	182	254	256	249	249	186	196	220	222	0	0	209	209	134	138
3267	Recunu (Bufania)	302	328	0	0	290	290	179	195	354	360	251	251	151	151	253	253	206	206	247	255	165	165	265	265	182	188	165	165	76	80	172	172	240	254	251	251	184	198	224	224	225	225	201	207	128	138
3269	Rucignola	302	318	0	0	294	294	185	191	352	370	249	251	149	151	247	247	206	206	249	249	165	183	263	263	186	188	165	179	74	80	188	206	240	254	241	245	194	200	224	224	227	227	209	209	138	140
3270	Russetto	318	322	233	233	288	294	179	179	356	358	0	0	149	149	247	247	206	214	243	251	165	187	263	263	186	186	163	163	74	80	178	188	240	254	0	0	0	0	0	0	0	0	191	209	138	140
3271	Sparo virdisi	316	322	257	257	294	294	179	189	352	360	249	251	149	149	247	247	206	210	243	243	169	183	255	263	176	188	165	165	74	74	166	186	238	238	243	251	186	194	224	238	233	241	191	209	128	140
3272	Sultanina	314	314	257	267	290	294	193	193	364	366	249	271	149	151	247	253	206	214	243	253	165	183	255	263	186	188	165	165	74	80	180	186	238	248	243	253	186	186	220	222	229	237	211	211	128	148
3273	Tallone nero	314	318	241	241	290	294	185	195	352	360	255	271	149	149	247	247	206	214	251	253	165	165	255	263	186	188	163	163	76	78	166	188	248	254	249	255	184	186	222	222	237	239	209	211	130	148
3274	Tintorè (Ibisu)	314	318	0	0	290	294	0	0	352	352	249	271	149	149	247	253	206	210	243	247	165	183	263	263	182	182	163	163	76	80	172	206	238	240	245	249	186	188	214	224	0	0	193	211	132	132
3275	Triboti nera	316	318	243	243	290	290	179	193	358	384	249	271	149	149	247	253	204	210	243	255	165	171	263	263	188	188	163	163	74	76	180	188	244	248	243	253	188	202	222	222	227	233	191	207	146	150
3276	Tripolina bianca	318	328	243	257	290	294	179	191	352	358	249	271	149	151	247	253	206	206	247	267	169	183	263	263	186	188	165	165	76	80	188	206	240	254	241	245	186	192	224	224	227	235	207	211	138	140
3277	Uva francisi	318	318	235	255	290	290	181	189	334	356	239	271	147	151	225	247	204	210	243	249	165	183	253	263	178	178	161	161	74	80	164	178	240	240	0	0	188	202	222	222	225	225	207	207	128	140
3278	Visparola	312	314	235	247	290	294	185	193	352	352	255	271	149	149	241	247	206	214	243	243	165	181	263	263	176	188	161	161	74	80	166	172	238	240	247	247	188	194	222	222	227	233	191	209	128	128
3279	Vitraruolo	302	318	0	0	294	294	0	0	356	358	251	251	151	157	241	247	206	210	0	0	183	183	263	263	186	186	161	165	76	80	170	172	248	254	243	243	200	200	224	224	0	0	201	209	128	138
3004	Zibibbo A	314	318	243	267	290	294	179	193	370	384	263	271	147	149	253	263	210	210	253	255	165	183	263	263	186	190	165	165	74	74	180	206	248	248	247	255	184	202	222	222	229	233	191	211	128	146
3280	Zibibbo augustano	314	318	241	265	290	294	179	193	370	384	251	263	147	149	255	263	210	210	0	0	165	183	263	263	186	190	165	165	74	74	180	206	248	248	243	251	184	200	222	222	227	231	191	211	128	154
3281	Zibibbo B	314	318	235	243	290	294	179	193	352	370	263	271	147	149	253	263	210	210	243	253	165	183	263	263	186	190	165	165	74	74	172	206	248	248	247	255	184	202	222	222	227	233	191	211	128	146
3282	Zibibbo carricante	314	318	243	267	290	294	179	193	352	352	263	271	147	149	253	263	210	210	253	251	165	183	263	263	186	190	165	165	74	74	172	206	248	248	247	255	184	202	222	222	229	233	191	211	128	146
3283	Zibibbo grosso	314	318	243	267	290	294	179	193	370	384	251	251	147	149	253	263	210	210	251	265	165	183	263	263	186	190	165	165	74	72	180	206	248	248	243	247	0	0	222	222	229	233	191	211	128	146
3284	Zibibbo nero	314	318	0	0	290	294	179	193	352	352	263	271	147	149	253	263	210	210	253	251	183	183	263	263	186	188	165	165	74	80	172	206	238	248	247	255	184	202	222	222	225	225	191	211	128	148
3285	Nero d'Avola B	314	314	0	0	290	294	179	181	352	352	251	251	147	149	247	253	206	210	243	253	183	183	263	263	186	188	163	163	80	78	172	178	0	0	251	251	184	200	222	222	0	0	209	209	138	148
3286	Nero d'Avola C	314	314	235	235	290	298	179	179	354	366	251	251	147	149	247	253	206	210	243	253	169	183	263	263	174	188	165	165	80	80	186	188	240	254	247	247	184	200	222	222	225	229	209	211	138	148
3287	Nero d'Avola D	314	318	243	243	294	306	181	185	356	370	251	271	149	151	247	247	206	206	243	257	169	179	263	263	180	188	165	173	74	76	182	206	254	254	251	259	184	198	224	224	227	229	209	209	132	138
3288	Racina i mustu A	318	318	235	243	290	298	179	185	352	354	251	255	149	151	239	239	206	212	243	253	165	169	263	263	174	188	165	165	80	80	172	172	240	254	241	247	192	200	222	222	237	241	211	213	128	138
3289	Cuda i vulpe	314	318	235	257	290	294	179	185	352	362	239	251	151	155	247	247	206	214	253	257	183	187	263	263	174	194	165	165	80	80	166	172	240	254	237	251	186	200	222	222	227	235	209	209	130	138
3290	Pirricone raspo rosso	318	324	241	241	294	294	185	185	352	366	251	271	149	149	247	247	206	210	249	255	165	173	255	263	186	186	163	163	74	76	166	206	238	254	239	251	186	202	222	222	225	237	191	211	128	130
3291	Malvasia A	318	328	233	253	290	294	179	183	348	362	251	271	149	155	247	247	206	214	243	249	165	185	257	263	172	182	157	163	76	80	172	182	240	254	241	245	186	200	224	224	225	225	191	209	138	142
3292	Nero d'Avola F	314	314	235	235	298	298	179	181	354	366	251	251	147	149	247	253	206	210	243	253	169	183	263	263	174	188	165	165	80	78	186	188	240	254	247	247	184	200	222	222	227	227	209	211	138	148
3293	Nerello Mascarisi	318	328	257	257	290	290	183	185	352	352	251	257	149	155	241	247	206	206	243	241	165	165	263	263	190	190	165	165	74	76	178	178	240	254	241	245	186	194	214	224	229	237	191	191	128	130
3294	Nero d'Avola G	314	314	235	235	298	298	179	181	354	366	251	251	147	149	247	253	206	210	243	253	169	183	263	263	174	188	165	165	80	78	186	188	240	254	251	251	184	200	222	222	227	241	209	211	138	148
3296	Cappuccio	314	318	227	233	290	294	189	189	0	0	257	257	147	149	253	263	210	210	251	265	165	183	263	263	0	0	165	165	74	74	180	206	238	254	249	251	192	194	214	224	0	0	191	211	128	146
3297	Perricone B	314	314	231	245	290	306	183	185	352	354	257	271	149	151	241	247	206	206	243	251	165	169	263	263	186	190	165	177	76	74	188	188	238	244	247	251	186	196	224	224	241	241	209	209	132	132

Supplementary Table S4. Genetic profiles of cultivated and wild Sicilian accessions.

Accession code	Population / Sample name	VVIp60		VVMD28		VVIb01		VVMD27		VVIv67		VVMD32		VVIn16		VVMD21		VVMD24		VVMD7		VMC1b11		VVIn73		VMIp31		VVIh54		VVIq52		VMC4f3_1		VVMD25		VrZag79		VrZag62		VVMD17		VVMD5		VVMD6		VVS2	
3298	Giugnatica	0	0	231	255	290	290	185	189	356	356	239	239	157	157	247	263	206	210	243	251	173	173	263	263	164	180	163	167	0	0	178	178	240	240	251	259	192	202	214	214	229	237	0	0	0	0
3299	Nero d'Avola E	314	314	235	241	298	298	179	181	354	366	249	251	147	149	247	253	206	210	243	253	169	183	263	263	174	188	165	165	78	80	186	188	240	254	247	247	184	200	222	222	227	227	209	211	138	148
3300	Putrisa	314	320	247	259	290	294	181	185	346	360	249	257	149	151	245	247	206	210	243	257	181	183	263	263	172	188	165	175	80	80	166	186	240	254	247	255	188	188	222	222	227	239	201	201	128	128
3302	Mantonico B	314	318	243	257	290	294	179	193	0	0	255	255	149	149	241	255	212	214	243	253	165	167	257	263	172	186	165	177	76	80	166	178	240	248	247	247	192	192	214	222	227	233	191	209	128	128
3304	Alicante, Licante	318	318	241	241	288	290	193	193	352	358	241	249	151	157	241	247	208	214	243	247	187	193	255	263	174	182	163	167	74	80	170	186	240	254	257	257	186	186	214	238	227	241	209	209	132	142
3305	Mantonico C	0	0	227	227	290	294	179	189	356	360	0	0	151	151	247	255	206	206	243	257	169	183	255	263	182	188	163	165	0	0	188	188	240	254	243	247	194	200	222	222	227	233	0	0	0	0
3307	Inzolia imperiale B	314	314	0	0	290	294	185	185	352	352	257	271	149	149	255	255	206	206	253	251	187	187	263	263	172	182	165	177	74	76	172	206	238	248	243	251	184	186	224	224	225	225	209	209	128	128
3308	Cantaro	314	316	241	255	290	294	179	179	352	384	249	271	149	151	241	253	206	206	235	249	169	187	263	263	190	190	165	175	74	76	182	188	238	244	245	245	186	200	220	224	233	245	209	211	128	138
3309	Trummana	316	318	0	0	290	294	179	179	356	358	249	251	149	149	247	253	204	210	243	241	165	171	263	263	186	188	165	165	74	74	178	188	240	254	245	249	186	200	224	224	225	225	191	191	148	134
3310	Minutidda	318	318	233	243	288	290	179	185	352	352	251	255	149	149	241	247	206	212	243	265	165	165	263	263	190	194	165	173	74	80	172	178	240	240	243	257	186	192	214	222	227	237	191	209	128	128
3311	Malvasia B	318	328	233	253	290	294	179	183	348	362	251	271	149	155	245	245	206	214	243	249	165	183	257	263	172	182	157	163	76	80	172	182	240	254	239	243	186	200	224	224	225	225	191	209	138	140
3312	Livedda	318	318	235	247	290	294	181	193	352	356	239	251	147	147	247	253	206	206	237	243	173	183	263	263	180	182	163	163	74	80	186	188	248	254	0	0	186	194	224	224	227	229	211	211	132	148
3313	Mascarisi	318	328	233	257	290	290	183	185	352	352	251	257	149	155	241	247	206	206	243	243	165	165	263	263	190	190	165	165	74	76	178	190	240	254	241	245	186	194	214	222	229	237	191	191	128	130
3314	Racina i mustu B	314	318	233	235	290	290	179	189	360	360	0	0	147	151	247	255	210	210	251	257	177	183	255	263	178	186	165	175	74	76	172	172	238	248	245	251	194	198	222	222	227	229	191	209	128	140
3315	Nuciddara	314	318	241	241	290	294	185	185	352	352	251	251	147	147	247	247	206	210	253	251	165	169	263	263	174	188	165	179	76	80	172	178	248	254	237	243	198	200	222	222	241	241	207	207	138	138
3316	Moscato nero	314	318	235	243	294	294	179	185	366	384	269	271	149	155	247	253	210	210	251	253	165	171	263	263	178	186	165	165	74	78	172	206	248	254	239	255	184	190	222	222	233	239	209	211	130	146
3317	Diretta nera B	318	318	235	243	290	298	179	193	352	358	251	251	149	151	247	253	206	210	243	253	165	183	263	263	174	188	165	165	76	80	188	206	240	254	251	251	184	200	222	222	227	233	209	211	132	138
3319	Minnilottina	314	318	243	247	0	0	185	193	368	370	251	251	149	151	247	253	206	206	237	243	179	183	263	263	0	0	0	0	76	80	206	206	238	238	251	251	184	200	222	222	229	239	211	213	132	134
3320	Mantonico A	318	322	243	243	294	294	185	185	352	366	251	271	149	149	247	247	206	210	253	259	165	173	255	263	186	186	163	165	74	76	166	206	238	254	239	251	184	202	220	220	227	239	191	211	128	130
3321	Cornicchiola A	312	314	243	257	290	290	179	181	342	352	257	261	147	151	247	263	206	210	251	253	165	183	263	263	180	194	159	165	80	84	166	202	238	248	251	251	194	202	222	224	237	249	191	211	140	146
3322	Lacrime i Maria B	316	318	241	241	290	290	179	195	358	384	0	0	149	149	247	255	204	210	239	249	165	171	263	263	188	190	163	163	74	76	180	188	244	248	0	0	0	0	0	0	223	229	191	209	146	150
3323	Inzolia nera A	312	314	231	245	290	306	169	183	352	354	0	0	149	151	241	247	206	206	241	249	165	169	263	263	186	190	165	177	76	80	188	190	238	244	243	247	186	196	224	224	239	239	209	211	128	152
3324	Fiore d'arancio (Trunzu)	314	328	235	243	290	294	179	185	352	352	271	271	151	151	253	263	206	206	237	243	165	169	263	263	174	184	165	175	74	80	188	206	248	254	251	257	194	202	222	222	227	233	191	211	128	148
3325	Lugliatica (Luglienga)	318	328	233	257	290	290	183	185	352	352	251	257	149	155	241	247	206	206	243	243	165	165	263	263	190	190	165	165	74	76	178	190	240	254	243	247	186	194	214	224	229	237	191	191	128	130
3326	Lacrime i Maria C	318	318	243	247	290	306	185	193	368	384	249	251	149	151	247	253	206	210	237	243	165	183	263	263	188	188	167	175	76	80	188	206	248	254	251	257	186	202	224	224	227	241	209	211	132	146
3327	Greca	314	318	247	257	290	306	185	185	356	366	251	257	149	149	247	253	206	206	237	243	183	183	263	263	188	200	165	175	74	80	202	206	248	254	247	253	188	202	222	224	239	239	209	211	132	138
3328	Caleu	314	322	257	257	290	294	183	193	352	356	261	271	149	151	247	253	206	206	237	247	183	183	263	263	182	188	165	165	76	80	186	188	238	248	247	257	186	202	224	224	239	241	209	211	128	148
3329	Minna di vacca C	318	318	0	0	290	294	179	185	352	358	239	251	149	149	253	253	204	206	243	247	165	183	263	263	188	190	163	163	76	80	188	202	248	254	0	0	186	194	224	224	233	239	207	211	132	146
3333	Funcia chiatta	314	318	241	241	290	290	179	193	358	366	249	271	149	151	251	253	210	214	243	241	165	183	263	263	174	190	165	167	74	80	166	188	248	254	251	257	184	186	222	222	227	227	209	209	138	146
3334	Bertuccio	314	322	0	0	290	294	181	185	0	0	249	251	149	151	247	251	206	212	243	243	173	183	263	263	176	188	165	165	74	76	178	178	240	254	251	259	184	186	220	220	225	225	207	207	138	140
3335	Catarratto E	0	0	227	235	290	294	179	179	352	368	251	251	149	149	247	253	206	210	243	253	165	183	263	263	174	178	165	165	0	0	0	0	240	254	243	247	194	198	222	222	227	227	0	0	0	0
3337	Inzolia nera B	314	320	243	257	290	294	185	193	356	368	239	261	0	0	0	0	0	0	243	257	167	183	263	263	188	188	165	175	76	80	166	186	238	248	247	247	192	198	222	222	233	233	203	211	128	128
3338	Alivedda nera	314	314	243	243	290	290	181	181	0	0	257	261	0	0	247	263	206	210	251	249	0	0	263	263	180	188	159	165	80	84	178	188	238	248	251	251	194	202	222	224	237	237	191	209	128	140
3339	Damaschino B	314	318	243	267	290	294	179	193	370	384	263	271	147	149	253	263	210	210	253	251	165	183	263	263	186	190	165	165	74	80	180	206	248	248	247	255	184	202	222	222	229	233	191	209	128	128
3340	Albanella bianca	318	318	227	227	290	294	179	189	352	356	251	271	151	151	247	253	206	206	243	257	169	183	255	263	186	188	165	177	74	80	186	188	238	248	247	251	194	200	222	222	233	233	211	211	132	148
3342	Menna vacca A	318	318	233	243	290	294	179	193	366	370	251	271	155	157	247	253	206	210	247	251	171	183	263	263	178	186	163	163	78	80	172	206	238	248	243	251	190	202	222	222	233	239	207	211	128	146
3343	Zu Manuele	0	0	0	0	290	294	179	191	352	360	255	261	157	157	247	255	206	210	243	253	181	183	263	263	188	190	163	163	80	78	188	206	240	248	247	249	184	202	224	224	225	225	209	209	0	0
3344	Centoruotoli	0	0	243	247	290	306	185	193	366	384	249	251	149	151	247	253	206	210	237	243	165	183	263	263	188	188	167	175	0	0	188	206	248	254	247	253	186	202	222	222	225	241	0	0	0	0
3345	Cornicchiola B	314	314	243	257	290	290	179	181	342	352	257	261	147	151	247	263	206	210	251	253	165	183	263	263	180	194	159	165	80	84	166	202	238	248	251	251	194	202	222	224	237	247	191	211	140	146
3348	Cutrera	324	322	233	233	290	290	187	193	352	352	251	271	149	149	247	253	206	210	243	251	165	169	255	263	182	190	165	165	74	74	172	188	238	238	251	257	190	198	222	222	229	229	209	211	128	138
3349	Zu Matteo	314	318	235	243	290	294	179	185	352	352	257	257	149	151	253	253	206	206	243	253	165	169	263	263	186	188	165	175	74	80	188	206	0	0	257	257	186	200	222	222	225	233	211	213	130	138
3350	Catanese bianca	314	328	231	245	290	298	179	183	354	370	249	255	151	155	241	247	206	206	247	249	165	187	263	263	188	188	165	165	74	76	172	190	238	254	245	257	188	192	224	224	225	241	211	211	128	148
3351	Dunnuni	314	318	0	0	290	294	179	185	370	384	0	0	147	149	253	263	206	210	243	253	165	183	263	263	178	188	165	165	74	80	180	206	248	254	0	0	186	186	222	222	0	0	207	209	128	138
3352	Fumusa	318	318	0	0	290	298	179	179	352	358	251	251	151	155	247	253	206	206	243	253	165	183	263	263	186	188	165	177	74	80	188	188	238	254	243	251	186	200	222	222	0	0	211	211	132	148
3354	Nocera	314	318	0	0	288	290	0	0	352	352	251	255	147	149	247	253	206	212	243	243	169	183	263	263	186	186	163	175	74	76	166	178	240	240	249	251	186	192	214	224	227	233	209	209	128	128
3355	Prunesta	314	318	227	233	290	294	179	179	360	360	0	0	149	149	247	253	206	210	247	247	165	169	263	263	182	190	165	165	74	80	172	188	240	248	251	255	186	202	222	222	225	225	207	211	128	144
3361	Inzolia Tunisina A	318	318	0	0	290	306	185	193	0	0	249	251	149	151	247	253	206	210	237	243	165	183	263	263	188	188	167	175	76	80	188	206	248	254	251	257	186	202	224	224	225	227	209	211	132	146
3362	Nero nostrale	318	328	227	233	290	294	183	183	352	352	251	257	0	0	241	247	206	206	243	243	165	165	263	263	176	188	165	165	74	76	178	188	238	238	243	247	186	194	224	224	229	229	191	191	128	130
3363	Zibibbo con i seni	314	318	241	265	290	294	179	193	370	384	263	271	147	149	253	263	210	210	249	251	165	183	263	263	186	190	165	165	74	72	180	206	248	248	245	251	184	202	222	222	227	231	191	211	128	146
3364	Garignano	0	0	247	257	290	294	181	185	356	370	249	251	149	151	247	251	206	206	243	243	165	183	263	263	174	182	165	165	0	0	178	186	238	254	249	255	184	186	222	222	225	227	0	0	0	0
3365	Zibibbo minna di vacca	314	318	227	247	290	294	179	179	352	352	263	263	149	149	263	263	210	210	253	251	165	165	263	263	174	188	163	165	74	74	178	188	238	254	247	255	184	202	222	222	0	0	191	191	128	146
3366	Zibibbo masculune	314	318	243	267	288	294	179	193	358	358	0	0	147	149	253	263	210	210	253	255	165	183	263	263	178	186	163	165	74	74	178	206	238	248	0	0	0	0	0	0	233	233	191	209	128	128
3367	Pionastro	318	318	247	247	290	294	183	193	352	360	251	255	149	151	247	253	206	206	243	253	183	183	263	265	182	190	165	165	74	80	188	206	254	266	247	257	190	202	224	224	233	239	191	211	130	144
3368	Inzolia Tunisina B	314	318	243	257	290	294	181	189	360	368	255	271	149	151	247	253	206	208	247	253	183	187	255	263	188	190	163	165	74	76	172	186	254	254	239	243	188	202	224	224	237	241	209	213	130	138
